# Habit and Automaticity in Medical Alert Override: Cohort Study

**DOI:** 10.2196/23355

**Published:** 2022-02-16

**Authors:** Le Wang, Kim Huat Goh, Adrian Yeow, Hermione Poh, Ke Li, Joannas Jie Lin Yeow, Gamaliel Tan, Christina Soh

**Affiliations:** 1 City University of Hong Kong Hong Kong China (Hong Kong); 2 Nanyang Technological University Singapore Singapore; 3 Singapore University of Social Sciences Singapore Singapore; 4 Medical Informatics National University Health System Singapore Singapore; 5 Ng Teng Fong General Hospital Singapore Singapore

**Keywords:** alert systems, habits, electronic medical record, health personnel alert fatigue

## Abstract

**Background:**

Prior literature suggests that alert dismissal could be linked to physicians’ habits and automaticity. The evidence for this perspective has been mainly observational data. This study uses log data from an electronic medical records system to empirically validate this perspective.

**Objective:**

We seek to quantify the association between habit and alert dismissal in physicians.

**Methods:**

We conducted a retrospective analysis using the log data comprising 66,049 alerts generated from hospitalized patients in a hospital from March 2017 to December 2018. We analyzed 1152 physicians exposed to a specific clinical support alert triggered in a hospital’s electronic medical record system to estimate the extent to which the physicians’ habit strength, which had been developed from habitual learning, impacted their propensity toward alert dismissal. We further examined the association between a physician’s habit strength and their subsequent incidences of alert dismissal. Additionally, we recorded the time taken by the physician to respond to the alert and collected data on other clinical and environmental factors related to the alerts as covariates for the analysis.

**Results:**

We found that a physician’s prior dismissal of alerts leads to their increased habit strength to dismiss alerts. Furthermore, a physician’s habit strength to dismiss alerts was found to be positively associated with incidences of subsequent alert dismissals after their initial alert dismissal. Alert dismissal due to habitual learning was also found to be pervasive across all physician ranks, from junior interns to senior attending specialists. Further, the dismissal of alerts had been observed to typically occur after a very short processing time. Our study found that 72.5% of alerts were dismissed in under 3 seconds after the alert appeared, and 13.2% of all alerts were dismissed in under 1 second after the alert appeared. We found empirical support that habitual dismissal is one of the key factors associated with alert dismissal. We also found that habitual dismissal of alerts is self-reinforcing, which suggests significant challenges in disrupting or changing alert dismissal habits once they are formed.

**Conclusions:**

Habitual tendencies are associated with the dismissal of alerts. This relationship is pervasive across all levels of physician rank and experience, and the effect is self-reinforcing.

## Introduction

### Background and Significance

Electronic medical records (EMR) systems have many embedded clinical support alerts to help warn or remind clinicians about patient-related issues [1]. However, the ubiquitous use of such alerts has led to a significant number of alert dismissals [2-8]. Some of these alerts were wrongly dismissed, leading to significant health consequences [1,9]. For example, Slight et al [10] estimated that in the United States alone about 57.6 million adverse drug event alerts were dismissed in 2014, and of those, about 5.5 million were inappropriately overridden, resulting in approximately 196,600 adverse drug events.

Prior literature suggests at least 3 interrelated reasons for the excessive dismissal of alerts. The first reason is the relevance or effectiveness of the alerts [11,12]. Physicians are more likely to dismiss less relevant alerts when they are repeatedly exposed to them. As such, studies have examined how the tiering of alerts based on their medical significance [13] could improve a physician’s alert compliance. Irrelevant alerts are also linked to the second reason—alert fatigue. Alert fatigue is a result of alert overload that causes clinicians to become desensitized to subsequent alerts [14,15]. The third reason, as suggested by Baysari et al [16], alerts are excessively dismissed is because of physicians’ habitual dismissal of alerts. Using field observations and physician interview data, the authors found that physicians had developed the habit of dismissing alerts over time, which resulted in an excessive number of alerts being dismissed without significant cognitive considerations. As such, Baysari et al [16] called for more empirical studies to examine the role that habit plays in influencing alerts dismissal and establish the prevalence of such incidences of habitual dismissal. In particular, they suggested studying the association between the number of alerts clinicians experience and their alert dismissal rates and how the rate of alert exposure impacts the formation of alert dismissal habits.

Put together, if physicians under high workload environments rely on habituated responses as a way to cope with alert overload and not critically process these alerts as a result [16], then it would have significant implications on the efficacy of alerts in situations that matter most. Therefore, we concur with Baysari et al [16] that it is important to establish the prevalence of such habitual behaviors among physicians and understand how such habits are formed and their impacts on patient care.

Habits are driven by an environmental stimulus that leads to consistent follow-up action in response to that stimulus. Quantifying habitual behavior using empirical observations is challenging; however, this area of research has received increased attention in recent years with the development of quantitative models to measure habit strength [17,18]. These models permit researchers to empirically quantify habitual actions to answer the call for empirically investigating the effects of habits on alert dismissal [16].

### Objective

The objective of this study is to empirically quantify the prevalence of alert dismissal associated with physician habit (habitual dismissal) as well as the association between a physician’s habitual dismissal and their subsequent tendency for alert dismissal.

## Methods

### Research Context

The study is a retrospective analysis of log data from 66,049 alerts generated from hospitalized patients captured by the Epic EMR system (Epic Systems Corp) over a 20-month period from March 2017 to December 2018.

### Settings and Data Context

**Figure 1:**
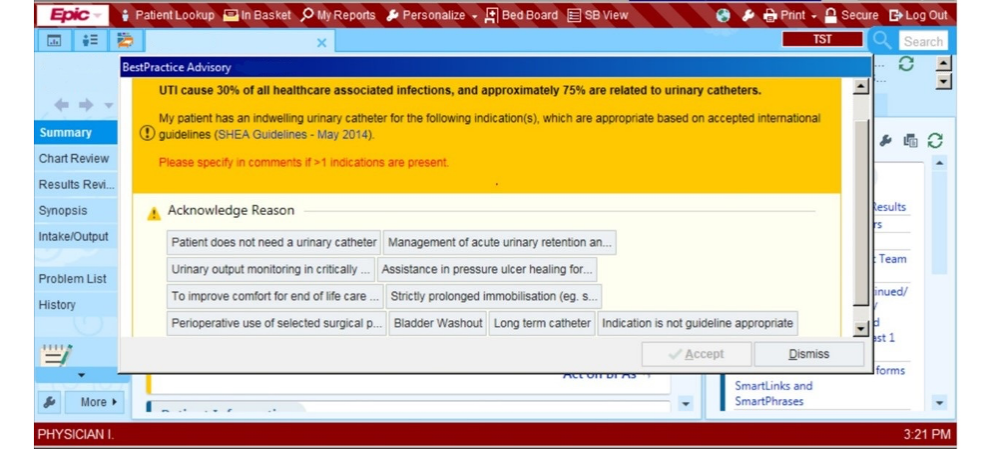
Indwelling catheter alert interface screenshot (modified to remove patient identifiers).

The study was situated at Jurong Health Campus, which consists of the integrated 700-bed Ng Teng Fong General Hospital and the 400-bed Jurong Community Hospital. An alert was set up in the EMR system to check if a patient had an indwelling catheter (IDC) for an extended period and was at risk of urinary tract infection. The IDC alert was activated each time the physician accessed the patient’s medical record, and it would have informed the physician of the following clinical guideline: “Urinary tract infection causes 30% of all health care–associated infections, and approximately 75% are related to urinary catheters.” A screenshot of the alert can be seen in Figure 1. The alert would have reminded the physician to assess the patient and indicate the reasons as to whether the patient should or should not continue to use the catheter. If the physician indicated that the catheter should be removed, they could enter the order in the Epic system. If the patient required continued use of the catheter, the physician could acknowledge this and indicate the reasons in the alert pop-up. In order to not obstruct the physician’s workflow, the alert also allowed the physician to ignore the alert by clicking on the dismiss button. Alerts that were acted upon by physicians (eg, alerts that were acknowledged with reasons for nonremoval or ordered for removal) would no longer appear. Alerts dismissed without acknowledgment continued to appear each time the physician accessed the patient’s medical record.

Between March 2017 and December 2018, we captured the actions of 1152 physicians who interacted with the IDC alert. This IDC alert was implemented in March 2017, after which we began the data capture. Physicians were not exposed to this alert prior to March 2017 as the IDC alert was custom-built and implemented only in this hospital campus. The IDC alert’s interface was also different from other clinical alerts in the EMR system as it is a customized alert. Our data consist of a total of 66,049 alerts from 1874 patients; each patient triggered an average of more than 30 alerts during their hospitalization. A total of 91.2% (60,236/66,049) of all alerts were dismissed by physicians.

### Outcome: Alert Dismissal

The unit of analysis for this study is each alert instance, and the main dependent variable is alert dismissal (*dismiss*), represented by a binary measure to indicate if the physician dismisses the alert. As a robustness check, we have also included 3 other possible dependent variables: dismissal performed in under 1, 2, or 3 seconds after the alert’s appearance (*dismiss1*, *dismiss2*, and *dismiss3*). We have included time limits for these alerts as a robustness check because habits are often associated with actions that are performed automatically with little cognitive effort and awareness.

Automaticity [16,19] is a trait of habits and an efficient way of handling familiar situations where the individual can execute actions with limited mental effort. Automaticity during a habitual action means that an individual is less likely to search for new information while ignoring additional situational information and will follow up with the usual habitual responses in a short amount of time [20]. Prior literature suggests that habitual dismissal occurs when physicians dismiss an alert without reading the content of the alert [16,21]. Given the clinical information included in the IDC alert, dismissal of alerts in under 1 second after they appeared were likely to be acts of automaticity and it was unlikely the physician had assessed the content of the alert. As a robustness check, other timing cutoffs were also used (ie, alerts dismissed in under 2 seconds [*dismiss2*] and 3 seconds [*dismiss3*]) with statistically similar findings.

### Primary Predictors: Habit Strength From Habitual Learning

To quantify habitual behavior, we represent habitual learning as a mathematical form of Hebbian learning as proposed in prior studies [17,22-26].

*H_t_*_+1_ = *H_t_* + *α_H_* (*α_t_* – *H_t_*) **(1)**

*H* represents the physician’s habit strength at different points in time denoted by *t*. α_t_ corresponds to the action taken on time *t*. An action that corresponded to the response observed in the habitual behavior (ie, dismissal of alert) was coded as 1; it was coded as 0 if the action was otherwise. α_H_ is a parameter that quantifies the rate of learning after each habitual action. Larger α_H_ values suggest a faster habit learning rate, and it typically takes on small values (ie, less than 1) [17]. For robustness and comparative purposes, this study used 3 values of α_H_ to compute the physician’s habit strength—0.01 (*H_0.01_*), 0.05 (*H_0.05_*), and 0.1 (*H_0.1_*). Given that the alert system is custom-built and that we captured all alert responses from the start of the alert’s implementation, we set the initial habit strength, *H_t_*, at 0 since no physicians had prior exposure to this particular alert before the implementation of the alert.

### Covariates

We divided the covariates used in this study into 5 categories: context of the alert, physician’s historical exposure to alerts, physician’s characteristics, patient’s characteristics, and timing effects. The names of the covariates are in brackets and italicized.

- Context of the alert: captured if the alert appeared during ward rounds (ward).
- Physician’s historical exposure to alerts were controlled for in 5 ways. First, given that many physicians can consult 1 patient and a physician can have many patients, we first computed the patient-physician (P_C) dyads. This dyad allowed us to precisely measure each physician’s alert exposure for a particular patient. We computed P_Ctotal, the total number of alerts a particular physician received for a particular patient. Second, we computed P_Cward, the total number of alerts received during ward rounds for the patient-physician pair. Third, to control for prior exposure to alerts unrelated to a particular patient, we computed the total number of alerts a particular physician received within the hospital from the beginning of the IDC alert program’s implementation (Ctotal). Physicians have heterogeneous experience and exposure to these alerts as they consult a large number of patients; the Ctotal variable controls for a physician’s familiarity with the system. Fourth, we controlled for the number of unique patients with indwelling catheters the particular physician encountered in a particular workday (CPatNum) to account for the physician’s exposure to patients with similar ailments. Finally, we controlled for the number of days since the physician,C, received the first alert for the patient, P.
- Physician characteristics: the variable C_rank (ie, seniority of the physician—intern, resident, fellow, or attending) was a proxy for the level of physician experience. We also captured the main medical specialty (specialty) in charge of the patient (eg, cardiology). Finally, we captured the type of ward the patient was in (dept).
- Patient characteristics: the patient’s age, gender, and race; the total length of stay (los) and number of diagnoses based on International Classification of Diseases, Tenth Revision classification (Diagnosis_count) were used as proxies for the severity of the patient’s condition.
- Timing effects: controlled for by recording the day of the week to account for the change in shift duty and the month of the year to account for seasonality.

The complete list of covariates and their detailed descriptions can be seen in Table S1 in Multimedia Appendix 1.

### Statistical Analysis

We computed the descriptive statistics to explore the distribution of how long it took each alert to be processed across different physician types and alert outcomes. We computed the point biserial correlations between the outcome variables and primary predictors, as well as Pearson correlations among the rest of the primary predictors. Calculating the point biserial correlation was required for instances where one of the variables was a binary variable.

To test the independent association of the physician’s habit strength on dismissal, we estimated 3 fixed effects, logistic regressions with the variable dismiss as the outcome variable, and each of the 3 measures of habit strength (*H_0.05_*, *H_0.01_*, and *H_0.1_*) as predictor variables. Fixed effects regression—grouped at the physician level—was required as the same physician’s dismissal behavior is likely to be correlated across alert instances, and controlling for physician-level effects allowed us to isolate the association between habit strength and dismissal outcomes beyond the physician’s idiosyncratic characteristics. As a robustness test, we estimated 3 more sets of fixed effects: logistic regression models using alternative, dependent variable measurements of dismissal (ie, *dismiss1*, *dismiss2*, and *dismiss3*). Likewise, the 3 primary predictors of habitual learning were regressed on each of these 3 alternative dependent variables, resulting in an additional (3 × 3) 9 regressions. As a further robustness check, we replicated the 12 regression models described above using regular logistic estimators and random effects logistic estimators for comparative purposes. These additional 24 models are reported in Multimedia Appendix 1.

All regressions used the covariates described above as controls during estimation, and we performed our analyses using Stata (version 14.2, StataCorp LLC).

### Ethics Approval

Ethics approval for this study was received by the Domain Specific Review Board (Ref: 2018/01306) in the National Healthcare Group, Singapore.

## Results

### Descriptive Statistics

Table 1 presents the summary statistics of our sample. We observed that physicians, on average, dismissed 91.2% (60,236/66,049) of all IDC alerts they encountered. Further, 13.3% (8750/66,049) of all alerts they encountered were dismissed in under 1 second, 56.9% (37,546/66,049) of alerts were dismissed in under 2 seconds, and 72.5% (47,890/66,049) of alerts were dismissed in under 3 seconds. A total of 45.6% (30,132/66,049) of all alerts appeared during the physician’s ward rounds (*ward*). Physicians, on average, were exposed to 84.9 alerts during this period in the hospital (*C_total_*). For any specific patient, a physician received, on average, about 3.9 alerts within the ward (*P_C_ward_*) and 8.2 alerts during the patient’s stay (*P_C_total_*). On any particular day, a physician would encounter an average of 1.3 patients (*C_PatNum_*) when the IDC alert was triggered.

**Figure 2:**
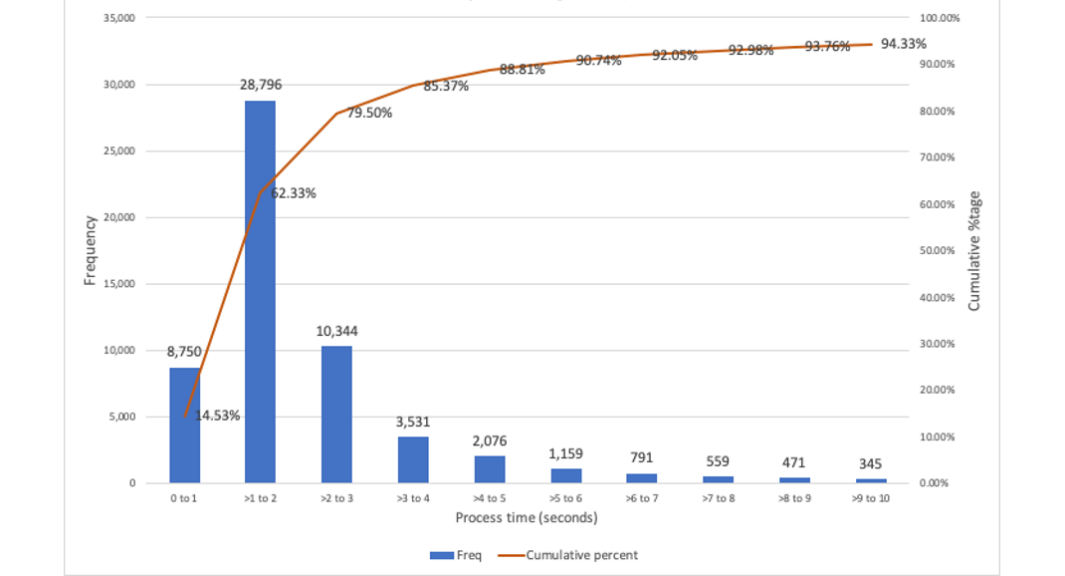
Distribution of processing time of all dismissed alerts.

Figure 2 presents the distribution of processing time for the alerts before they were dismissed. We observed that, on average, 79.5% (47,890/60,236) of all dismissed alerts were dismissed in under 3 seconds, and 14.5% (8750/60,236) of all dismissed alerts were dismissed in under 1 second.

**Figure 3:**
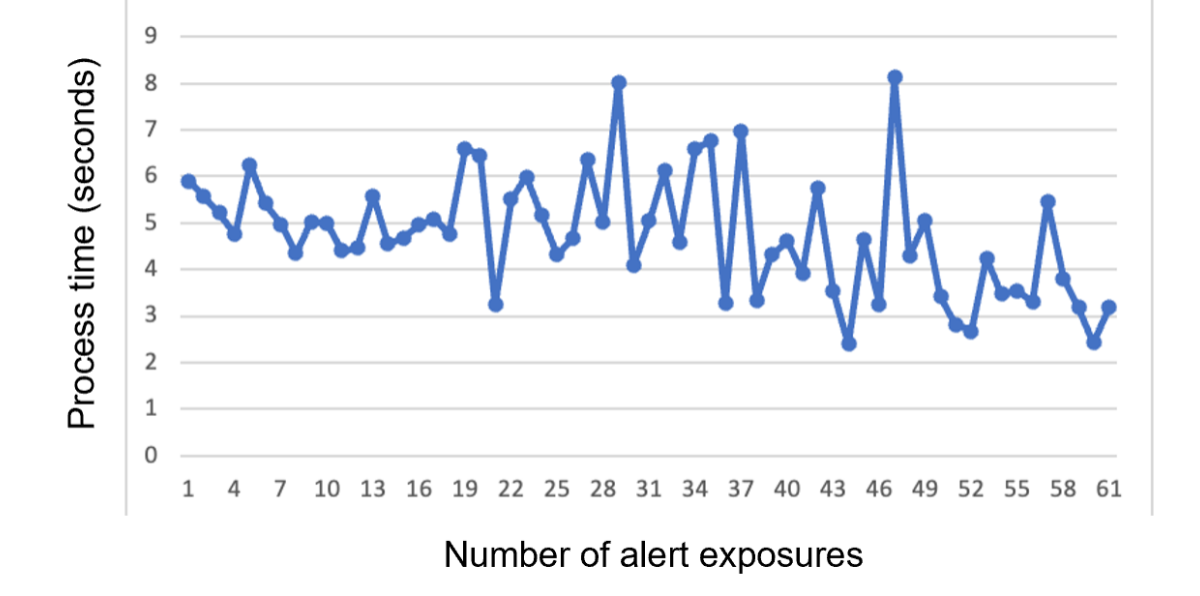
Physicians’ average alert processing times across number of alert exposures.

We also observed changes in physicians’ response times over time (Figure 3). The average time for a physician to process an alert dropped from 5.90 seconds (95% CI 5.46-6.34) in the first alert exposure to 2.43 seconds (95% CI 1.75-3.10) during the 60th exposure.

**Figure 4:**
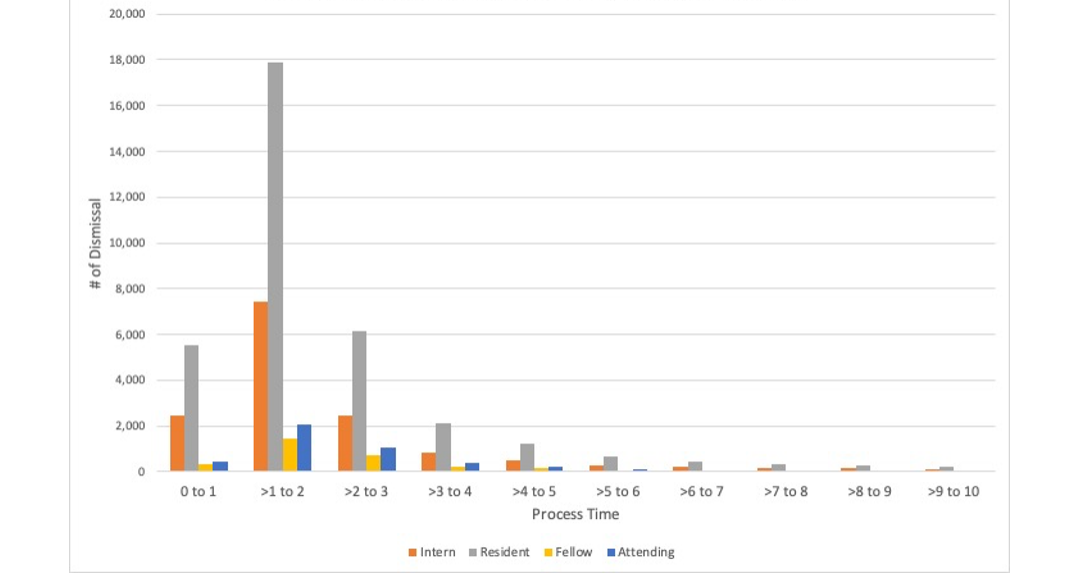
Distribution of response time by physician’s rank (level of experience).

We further explored the distribution of dismissal times across different physician experience levels (Figure 4) and observed similar patterns in dismissal times across medical interns, residents, fellows, and attending physicians. These results suggest that habitual tendencies in dismissing alerts apply to all levels of medical experience.

**Figure 5:**
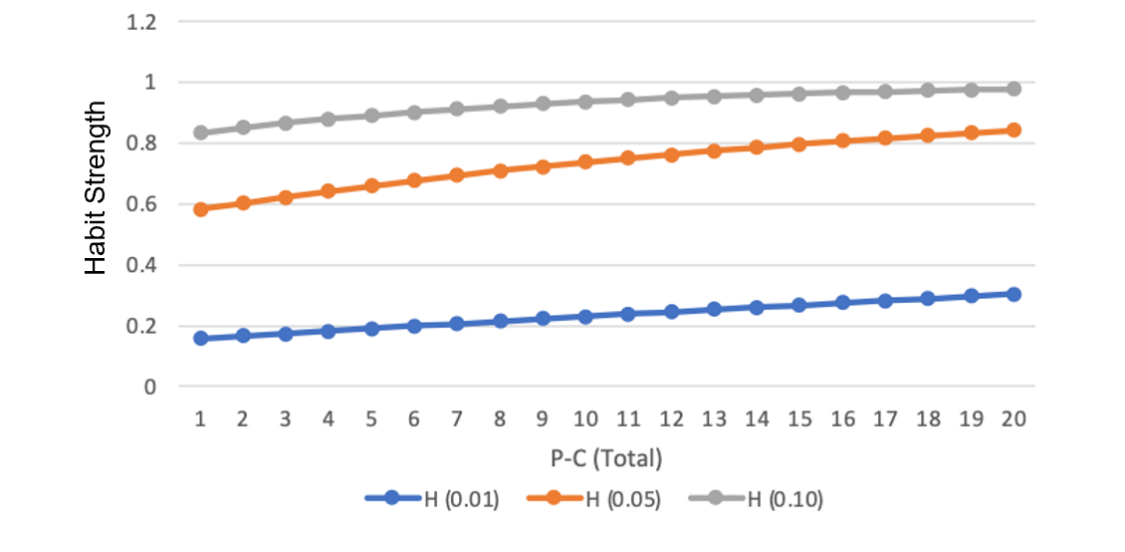
Variation of habit strength across number of alert exposures. Note: P-C (Total) represents the number of alert exposures a physician experiences.

To visualize how a physician’s habit strength (*H_0.01_*, *H_0.05_*, and *H_0.10_*) changed relative to their increasing exposure to the alerts, see the relationship plotted for a typical physician in Figure 5.

**Table 1:** Summary statistics of the variables^a^.

Variable	Mean (SD)	Minimum	Maximum
*dismiss*	0.912 (0.283)	0	1
*dismiss1*	0.132 (0.339)	0	1
*dismiss2*	0.568 (0.495)	0	1
*dismiss3*	0.725 (0.446)	0	1
*H_0.01_*	0.400 (0.292)	0	1
*H_0.05_*	0.708 (0.316)	0	1
*H_0.01_*	0.792 (0.291)	0	1
*ward*	0.456 (0.498)	0	1
*P_C*_total_	8.216 (11.961)	1	120
*P_C_ward_*	3.876 (5.991)	0	60
*C_total_*	84.868 (93.886)	1	669
*C_PatNum_*	1.303 (0.619)	1	7
*day_lag*	3.164 (8.826)	0	240
*age*	67.903 (16.125)	16	105
*gender*	0.558 (0.497)	0	1
*los*	38.425 (48.369)	1	357
*Diagnosis_count*	1.044 (0.234)	1	3

^a^All variables listed above were described earlier in the text. Refer to Table S1 in Multimedia Appendix 2 for a list of detailed definitions of the variables.

The pair-wise correlations between the outcome variable and primary predictors can be found in Table 2. Alert dismissal was found to positively correlate with all primary predictors (habit strength) in this study, with correlations ranging from 0.227 to 0.421 (*P*<.001). Further, all dismissals under 1, 2, and 3 seconds (*dismiss#*) were found to positively correlate with the physicians’ habit strengths. The point biserial correlation values ranged from 0.024 (*P*<.001) to 0.270 (*P*<.001). The correlations were found to be higher for dismissals under 2 seconds (the correlation values ranged from 0.106 to 0.220; *P*<.001) than dismissals under 1 second (the correlation values ranged from 0.024 to 0.082; *P*<.001). Dismissals under 3 seconds had the highest correlations between the physician’s habit strength and alert dismissal (correlations ranged from 0.136 to 0.270; *P*<.001). We observed stronger positive correlations for a higher rate of habitual learning with dismissal outcomes, indicating that physicians with stronger habitual tendencies were more likely to dismiss alerts within a short period of time.

**Table 2:** Correlations^a^ among outcome variables and key predictors.

	*dismiss* (*P* value)	*dismiss1* (*P* value)	*dismiss2* (*P* value)	*dismiss3* (*P* value)	*H_0.01_* (*P* value)	*H_0.05_* (*P* value)	*H_0.1_* (*P* value)
*dismiss*	.99	—^b^	—	—	—	—	—
*dismiss1*	0.121 (<.001)	.99	—	—	—	—	—
*dismiss2*	0.357 (<.001)	0.341 (<.001)	.99	—	—	—	—
*dismiss3*	0.505 (<.001)	0.241 (<.001)	0.707 (<.001)	.99	—	—	—
*H_0.01_*	0.227 (<.001)	0.024 (<.001)	0.106 (<.001)	0.136 (<.001)	.99	—	—
*H_0.05_*	0.347 (<.001)	0.065 (<.001)	0.183 (<.001)	0.223 (<.001)	0.845 (<.001)	.99	—
*H_0.01_*	0.421 (<.001)	0.082 (.002)	0.220 (<.001)	0.270 (<.001)	0.703 (<.001)	0.956 (<.001)	.99

^a^Variables *dismiss*, *dismiss1*, *dismiss2*, and *dismiss3* are binary, so we perform a point biserial correlation for their relationships with other predictors. All other correlations are Pearson correlations.

^b^Not applicable.

### Regression Results

The regression results in Multimedia Appendix 2 show the association between physicians’ current habit strength of alert dismissal (*H_0.01_*, *H_0.05_*, *H_0.1_*) and the probability of their dismissal of the current alert (*dismiss*). The results show that all 3 ways of quantifying habit strengths were positively associated with an increased likelihood of dismissing alerts received in the present time. A 1 standard deviation increase in habit strength was associated with an increase in odds of a physician’s dismissal of an alert they received in the present time by 0.642 to 0.810 times.

The results for the other 9 models with alternative measures of dismissals performed within 1, 2, and 3 seconds of appearance (*dismiss1*, *dismiss2*, and *dismiss3*) were also consistent with the main findings. Physicians with higher habit strength were found to more likely dismiss the next alert in under 1 to 3 seconds. Specifically, every 1 standard deviation increase in habit strength was associated with an increase in odds ratio by 0.362 to 0.510 times for the dismissal of the next alert within 1 second of the alert’s appearance. The effect was similar for dismissals that occur in under 3 seconds of the alert’s appearance—a 1 standard deviation increase in a physician’s habit strength was associated with an increase in odds ratio of 0.350 to 0.503 times for dismissing the subsequent alert. We further computed a random-effects estimator to derive the intraclass correlation coefficient for this model and found that physician characteristics account for 0.716 of the total unexplained variance in dismissal.

Extant studies have found mixed results on how physicians of different ranks (ie, level of experience) would respond to alerts differently. For example, Baysari et al [27] found that only 17% of alerts presented to senior doctors were read compared to junior doctors reading 78% of patient alerts they received. However, Straichman et al [28] and Tamblyn et al [29] found that a physician’s seniority had no impact on their alert overriding behavior. To explore if habits had a different influence on physicians of different ranks (levels of experience), we divided the alert instances into 4 subsamples based on the physician’s rank (intern, resident, fellow, or attending). For each subsample, we observed that an increase in habit strength was positively associated with dismissals, with dismissals below 1 to 3 seconds of alert exposure across all physician ranks (Table 3).

**Table 3:** Fixed effects logistic regression results for different physician ranks.

Physician rank and habit strength			*dismiss*					*dismiss1*					*dismiss2*					*dismiss3*		
			β^a^ (95% CI)		*P* value		β (95% CI)			*P* value		β (95% CI)			*P* value		β (95% CI)			*P* value
**Intern (first year post-medical school)**																				
	*H_0.05_*	3.235 (2.785-3.685)		.23		1.656 (1.368-1.944)			.15		1.476 (1.288-1.664)			.10		1.560 (1.364-1.756)			.10	
**Resident (2 to 6 years of work experience)**																				
	*H_0.05_*	2.604 (2.374-2.835)		.12		1.354 (1.174-1.535)			.10		1.496 (1.374-1.618)			.06		1.486 (1.357-1.614)			.07	
**Fellow (completed residency and in specialist training)**																				
	*H_0.05_*	1.591 (0.372-2.811)		.62		2.285 (1.397-3.173)			.45		1.831 (1.333-2.329)			.25		1.584 (1.010-2.157)			.29	
**Attending physician (specialists)**																				
	*H_0.05_*	1.830 (1.008-2.651)		.42		1.934 (1.272-2.596)			.34		2.077 (1.641-2.512)			.22		1.652 (1.184-2.121)			.24	

^a^Coefficients are exponentiated and represent odds ratios. Each cell represents the coefficient of a single fixed effects logistics regression. Full regression results are available in Multimedia Appendix 1. All multivariate models are adjusted for the context of the alert, physician’s historical exposure to alerts, physician characteristics, patient characteristics, and timing effects.

Finally, we computed the relationship between habit strength and physician processing time in an alternative analysis with fixed effects, random effects, and ordinary least square estimators. Here, we observed that processing time of alert is significant and negatively associated with habit strength after controlling for contextual factors, providing further evidence for the association between habit and automaticity (β_fixed_effects_=–1.161, *P*<.10; β_random_effects_=–1.305, *P*<.05; β_OLS_=–2.402, *P*<.05). We further computed a random-effects estimator to derive the intraclass correlation coefficient for this model and found that physician characteristics account for 0.164 of the total unexplained variance in processing time.

## Discussion

### Principal Findings

Physicians in clinical settings often experience high workload, significant time pressure, and information overload. The use of clinical support alerts supposedly ensures that, amid these challenging work conditions, physicians can quickly attend to patients potentially at risk of adverse events. The irony, however, is that the very same challenging conditions are also the conditions that may result in the formation of habits in physician responses to the alerts [16,20]. Under such conditions of high workload and information overload, individuals are more likely to rely on automaticity in their responses. Further, as physicians experience more alerts, as argued by Baysari et al [16], they may form habits that are biased toward more habitual dismissal. We empirically show that this is true in our study’s clinical context.

Our study contributes to the area of habit research by operationalizing an empirical measure of habitual actions. Traditionally, habitual actions are observed and understood from the perspective of the physician performing the action. However, it is inherently difficult to determine the cognitive state of the physician as they perform different actions [19]. As such, it is challenging to conduct a large-scale empirical study to quantify physician habits. Our study provides a different approach to address this challenge by using a theoretically based alternative that tracks repeated actions as an empirical proxy to quantify habitual actions [18]. We tested the habit strength measure’s relationship with the physician’s subsequent alert dismissal and found significant positive relationships between them. Our additional analyses also showed that habit strength measures yielded consistent and similar results with models that used simpler measurements of habit such as physicians’ past dismissal rates. Our empirical method of quantifying the habitual response of alerts provides a way for future empirical research that studies the role of habits in physician actions in general.

Our study also answers the call for more systematic empirical research to examine the role of habits in high alert dismissal. Our findings, where we observed a large proportion of alerts dismissed in under 3 seconds, provide empirical evidence of physicians relying on automaticity while dismissing alerts. Our findings support the view that habits play a significant role in influencing alert response.

Specifically, our findings provide 3 key insights into the relationship between habits and alerts. First, we found that the association between habit strength and alert dismissal is pervasive in the sample of alert responses we studied. Second, we showed that habitual alert dismissal occurs in physicians across all levels of experience; senior and junior physicians alike all have the tendency to habitually dismiss alerts. Together, these insights suggest that health informatics professionals and designers of clinical support alerts need to take into account the effect of habits on physicians when designing, implementing, and interpreting the impact of clinical alerts. Research on alert relevance that uses dismissal rates as a measure of relevance (ie, lower dismissal rates suggest a higher relevance of the alert) need to take into account how fast alerts are dismissed. Our study found that there is more to alert dismissal than simply a physician’s judgment of the alert’s relevance. This is especially true for alerts dismissed within time durations that are too brief to support any meaningful assessment of the alert. Third, we found that habits are self-perpetuating, self-reinforcing, and, as a result, hard to mitigate once they are formed. Physicians who have a history of dismissing alerts are more likely to dismiss subsequent alerts and tend to dismiss them more hastily without much consideration. The self-reinforcing nature of habitual dismissal is a cause of concern as it may significantly reduce the effectiveness of the focal alert and possibly impact the physicians’ responses to other clinical support alerts implemented in a clinical setting.

However, given our understanding of the role of a physician’s habit formation in the context of alert dismissal and the prevalence of habitual dismissal in health care settings, we propose that future research should draw on our understanding to examine how work conditions and alerts could be designed to mitigate the formation of habitual behavior. Extant literature informs us that habits are shaped by associating an external cue with a repeated, stable response, which, in turn, produces a set of consistent consequences [30]. Habits form when individuals experience a stable context that triggers a habitual action performed frequently, and the habitual action leads to an affirmative outcome that reinforces the behavior. These 3 antecedents are necessary for habit formation, and prior studies on strategies to reduce habitual responses have suggested disrupting or removing these antecedents to reduce habitual behaviors [31,32]. One example to mitigate habit formation is to disrupt the stable environment and triggers that lead to habitual responses. Although these studies are not situated in the medical context, the strategy of removing the individual’s familiar environment and triggers of habitual response is an approach that could be explored in the clinical alert context. For example, designers of clinical support alerts could consider varying the format and form of the alerts based on the risk levels or types of clinical conditions involved. Rare alerts associated with higher risks could be designed with a different alert interface, which could disrupt the stable environment (similar presentation mode) that triggers the habitual response. Furthermore, the alert interface could be programmed to be refreshed periodically to remove the stable environment required for habit formation and thereby reduce the incidence of habitual responses.

### Limitations

One limitation of this study is that it examined habitual dismissal for only one type of clinical alert: the removal of IDCs. Although we examined all the alerts that appeared during the sample time frame, future research could include different types of alerts as part of the study to understand if our findings are generalizable beyond a specific alert. However, studies involving different alerts will require these additional alert systems to be custom-built to accurately analyze physician responses from the start of the alert’s implementation to quantify physician habit strength. Studies that use existing alert systems may not yield accurate findings, as physicians would have had prior exposure to these alerts. Although we attempted to control for physicians’ alert fatigue by including, as a covariate, the number of unique patients with the IDC alert the physician consulted on that day, this may not completely control for alert fatigue that might result from other types of medical alerts. Again, future research may attempt to include the impact of other alerts as a control in the study.

Finally, like most retrospective cohort studies, our study does not seek to establish causal evidence or a causal relationship between a physician’s habits and the subsequent dismissal of alerts. Here, we use a computational model of habit to show the association between habit formation and a physician’s dismissal of alerts within a real-world clinical context. Without further studies that examine the intentions, less feasible in a real-world clinical context, the ability to isolate habit as the sole cause of dismissal is challenging and calls for further research.

### Conclusion

This study shows that the strength of a physician’s habit for dismissing medical alerts is positively associated with their tendency for subsequent incidences of alert dismissal. Additionally, it was found that most (72.5%) dismissed alerts occurred in under 3 seconds of the physician’s exposure to the alert. This empirical finding is in line with prior health informatics literature, suggesting the role of habit in EMR alert dismissal. We contribute to this stream of work by showing that habitual dismissal occurs across all levels of physician experience and that this is a self-reinforcing phenomenon. As physicians habitually dismiss alerts, the likelihood of them hastily dismissing subsequent alerts increases significantly. This phenomenon presents challenges to removing such inclinations toward habitual dismissal among physicians.

## Appendix

Multimedia Appendix 1 Supplementary tables.

Multimedia Appendix 2 Fixed effects logistics regression results for dismissal.

## Abbreviations

EMR: electronic medical record
IDC: indwelling catheter
