# Supplementary material for: Habit and Automaticity in Medical Alert Override: Cohort Study
Source: J Med Internet Res. 2022 Feb 16;24(2):e23355. doi: 10.2196/23355 (PMC8892274; doi:10.2196/23355)
Supplement: Multimedia Appendix 1 [file jmir_v24i2e23355_app1.docx]

**Multimedia Appendix 1: Supplementary tables.**

**Table S1. Definition of variables used**

| Variable | Definition |
| --- | --- |
| *dismiss* | Whether the alert was dismissed, a binary flag |
| *dismiss#* | Whether the alert was dismissed within # second(s) of appearance, a binary flag |
| *H_0.01_* | Habitual learning. With rate of learning, *α_H_* set at 0.01 |
| *H_0.05_* | Habitual learning. With rate of learning, *α_H_* set at 0.05 |
| *H_0.1_* | Habitual learning. With rate of learning, *α_H_* set at 0.1 |
| *Covariates* | |
| Variable | **Definition** |
| *ward* | Whether the alert appears during the ward rounds |
| *P_C_total_* | The number of alerts encountered by the physician for the particular patient |
| *P_C_ward_* | The number of alerts encountered by the physician for the particular patient during the ward round |
| *C_total_* | The total number of alerts encountered by the physician |
| *C_PatNum_* | The number of unique patients encountered by the physician on the day |
| *day_lag* | The number of days since the physician saw the first alert of the patient |
| *C_rank* | The level of physician experience |
| *age* | The age of the patient |
| *gender* | 0 for female, and 1 for male |
| *race* | Dummy variables for different races |
| *los* | The length-of-stay for the patient in this hospitalization encounter |
| *Diagnosis_count* | The number of diagnoses of the patients based on ICD-10 classification |
| *day* | Monday through Sunday as dummy variables |
| *dept* | The ward the patient is in. Different specialities have different wards |
| *spec* | The specialty of the physician who receives the alert |
| *mth* | Jan through Dec, the month in which the alert appears |

**Table S2. Fixed Effects Logistic Regression Models with Dismiss and Dismiss1 as Dependent Variables**

| Outcome  Predictor | dismiss | dismiss | dismiss | dismiss1 | dismiss1 | dismiss1 |
| --- | --- | --- | --- | --- | --- | --- |
|  | β coeff.  [95% C.I.]  (P value)  (std.error) | β coeff.  [95% C.I.]  (P value)  (std.error) | β coeff.  [95% C.I.]  (P value)  (std.error) | β coeff.  [95% C.I.]  (P value)  (std.error) | β coeff.  [95% C.I.]  (P value)  (std.error) | β coeff.  [95% C.I.]  (P value)  (std.error) |
|  |  |  |  |  |  |  |
| H_0.01_ | 2.195^***^ |  |  | 1.663^***^ |  |  |
|  | [1.767,2.623] |  |  | [1.376,1.949] |  |  |
|  | (0.000) |  |  | (0.000) |  |  |
|  | (0.220) |  |  | (0.147) |  |  |
| H_0.05_ |  | 2.506^***^ |  |  | 1.391^***^ |  |
|  |  | [2.312,2.700] |  |  | [1.231,1.551] |  |
|  |  | (0.000) |  |  | (0.000) |  |
|  |  | (0.100) |  |  | (0.082) |  |
| H_0.1_ |  |  | 2.784^***^ |  |  | 1.540^***^ |
|  |  |  | [2.620,2.948] |  |  | [1.379,1.701] |
|  |  |  | (0.000) |  |  | (0.000) |
|  |  |  | (0.085) |  |  | (0.083) |
| ward | -0.209^***^ | -0.209^***^ | -0.203^***^ | -0.126^***^ | -0.123^***^ | -0.120^***^ |
|  | [-0.287,-0.131] | [-0.288,-0.130] | [-0.283,-0.123] | [-0.179,-0.073] | [-0.176,-0.070] | [-0.173,-0.067] |
|  | (0.000) | (0.000) | (0.000) | (0.000) | (0.000) | (0.000) |
|  | (0.040) | (0.041) | (0.041) | (0.027) | (0.027) | (0.027) |
| P_C_total_ | 0.017^***^ | 0.008 | 0.005 | -0.000 | -0.001 | -0.002 |
|  | [0.004,0.030] | [-0.005,0.021] | [-0.007,0.018] | [-0.006,0.006] | [-0.007,0.005] | [-0.008,0.004] |
|  | (0.008) | (0.211) | (0.413) | (0.996) | (0.644) | (0.559) |
|  | (0.006) | (0.007) | (0.007) | (0.003) | (0.003) | (0.006) |
| P_C_ward_ | -0.037^***^ | -0.032^***^ | -0.031^**^ | 0.008 | 0.008 | 0.009 |
|  | [-0.060,-0.014] | [-0.056,-0.009] | [-0.054,-0.007] | [-0.004,0.020] | [-0.004,0.020] | [-0.003,0.021] |
|  | (0.002) | (0.007) | (0.012) | (0.170) | (0.168) | (0.146) |
|  | (0.012) | (0.012) | (0.012) | (0.006) | (0.006) | (0.006) |
| C_total_ | -0.000 | 0.002^***^ | 0.002^***^ | -0.004^***^ | -0.001^***^ | -0.001^***^ |
|  | [-0.002,0.001] | [0.001,0.002] | [0.002,0.003] | [-0.005,-0.003] | [-0.002,-0.001] | [-0.001,-0.000] |
|  | (0.475) | (0.000) | (0.000) | (0.000) | (0.000) | (0.009) |
|  | (0.001) | (0.000) | (0.000) | (0.000) | (0.000) | (0.000) |
| C_PatNum_ | -0.009 | -0.041 | -0.044 | 0.053^**^ | 0.044^**^ | 0.043^**^ |
|  | [-0.076,0.057] | [-0.108,0.026] | [-0.112,0.025] | [0.011,0.095] | [0.001,0.086] | [0.001,0.085] |
|  | (0.778) | (0.231) | (0.210) | (0.013) | (0.043) | (0.045) |
|  | (0.034) | (0.034) | (0.035) | (0.022) | (0.022) | (0.022) |
| day_lag | -0.007^***^ | -0.006^**^ | -0.006^**^ | -0.005^*^ | -0.005^*^ | -0.005^*^ |
|  | [-0.012,-0.002] | [-0.011,-0.001] | [-0.011,-0.001] | [-0.010,0.001] | [-0.010,0.000] | [-0.010,0.001] |
|  | (0.005) | (0.011) | (0.025) | (0.084) | (0.074) | (0.091) |
|  | (0.002) | (0.003) | (0.003) | (0.003) | (0.003) | (0.003) |
| age | -0.001 | -0.001 | -0.002 | 0.001 | 0.001 | 0.001 |
|  | [-0.003,0.002] | [-0.004,0.002] | [-0.005,0.001] | [-0.001,0.003] | [-0.001,0.003] | [-0.001,0.003] |
|  | (0.678) | (0.353) | (0.238) | (0.446) | (0.413) | (0.406) |
|  | (0.001) | (0.001) | (0.002) | (0.001) | (0.001) | (0.001) |
| gender | -0.094^**^ | -0.105^**^ | -0.106^**^ | -0.007 | -0.004 | -0.005 |
|  | [-0.176,-0.013] | [-0.188,-0.023] | [-0.190,-0.023] | [-0.066,0.051] | [-0.062,0.055] | [-0.064,0.053] |
|  | (0.023) | (0.013) | (0.013) | (0.804) | (0.906) | (0.863) |
|  | (0.042) | (0.043) | (0.043) | (0.030) | (0.030) | (0.030) |
| los | 0.001 | 0.001^*^ | 0.001^*^ | -0.004^***^ | -0.003^***^ | -0.003^***^ |
|  | [-0.000,0.002] | [-0.000,0.002] | [-0.000,0.003] | [-0.004,-0.003] | [-0.004,-0.003] | [-0.004,-0.002] |
|  | (0.143) | (0.059) | (0.055) | (0.000) | (0.000) | (0.000) |
|  | (0.001) | (0.001) | (0.001) | (0.000) | (0.000) | (0.000) |
| Diagnosis_count | 0.043 | 0.067 | 0.059 | -0.120^*^ | -0.112 | -0.109 |
|  | [-0.166,0.251] | [-0.142,0.277] | [-0.153,0.270] | [-0.261,0.021] | [-0.254,0.029] | [-0.250,0.032] |
|  | (0.686) | (0.528) | (0.586) | (0.094) | (0.118) | (0.130) |
|  | (0.107) | (0.108) | (0.109) | (0.072) | (0.072) | (0.072) |
| dept | Yes | Yes | Yes | Yes | Yes | Yes |
| spec | Yes | Yes | Yes | Yes | Yes | Yes |
| race | Yes | Yes | Yes | Yes | Yes | Yes |
| day | Yes | Yes | Yes | Yes | Yes | Yes |
| mth | Yes | Yes | Yes | Yes | Yes | Yes |
| constant | 2.923 | 2.622 | 2.432 | -14.983 | -15.518 | -15.743 |
|  | (1.832) | (1.853) | (1.871) | (862.434) | (846.736) | (849.299) |
| *N* | 49221 | 49221 | 49221 | 61583 | 61583 | 61583 |
| pseudo *R*^2^ | 0.362 | 0.378 | 0.391 | 0.140 | 0.143 | 0.145 |
| *AIC* | 23978.503 | 23429.971 | 22938.929 | 45010.867 | 44834.143 | 44752.286 |
| *BIC* | 30158.964 | 29610.433 | 29119.391 | 52738.955 | 52562.232 | 52480.375 |
| Log Likelihood | -11287.252 | -11012.986 | -10767.465 | -21649.433 | -21561.072 | -21520.143 |

**Note:** Coefficients are exponentiated and represent odds ratios. *H_#_* represents habits strength with # as the corresponding habit learning rate. All multivariate models adjusted for the context of the alert, physician's historical exposure to alerts, physician characteristics, patient characteristics, and timing effects. ^***^ indicates *p* < 0.01, ^**^ indicates *p* < 0.05, ^*^ indicates *p* < 0.10.

**Table S3. Fixed Effects Logistic Regression Models with Dismiss2 and Dismiss3 as Dependent Variables**

| Outcome  Predictor | dismiss2 | dismiss2 | dismiss2 | dismiss3 | dismiss3 | dismiss3 |
| --- | --- | --- | --- | --- | --- | --- |
|  | β coeff.  [95% C.I.]  (P value)  (std.error) | β coeff.  [95% C.I.]  (P value)  (std.error) | β coeff.  [95% C.I.]  (P value)  (std.error) | β coeff.  [95% C.I.]  (P value)  (std.error) | β coeff.  [95% C.I.]  (P value)  (std.error) | β coeff.  [95% C.I.]  (P value)  (std.error) |
| H_0.01_ | 1.236^***^ |  |  | 1.197^***^ |  |  |
|  | [1.054,1.419] |  |  | [0.994,1.400] |  |  |
|  | (0.000) |  |  | (0.000) |  |  |
|  | (0.093) |  |  | (0.104) |  |  |
| H_0.05_ |  | 1.467^***^ |  |  | 1.439^***^ |  |
|  |  | [1.364,1.569] |  |  | [1.331,1.547] |  |
|  |  | (0.000) |  |  | (0.000) |  |
|  |  | (0.052) |  |  | (0.055) |  |
| H_0.1_ |  |  | 1.753^***^ |  |  | 1.728^***^ |
|  |  |  | [1.653,1.852] |  |  | [1.627,1.829] |
|  |  |  | (0.000) |  |  | (0.000) |
|  |  |  | (0.051) |  |  | (0.052) |
| ward | -0.198^***^ | -0.196^***^ | -0.192^***^ | -0.208^***^ | -0.205^***^ | -0.201^***^ |
|  | [-0.235,-0.160] | [-0.234,-0.158] | [-0.230,-0.155] | [-0.250,-0.167] | [-0.247,-0.164] | [-0.243,-0.159] |
|  | (0.000) | (0.000) | (0.000) | (0.000) | (0.000) | (0.000) |
|  | (0.019) | (0.019) | (0.019) | (0.021) | (0.021) | (0.021) |
| P_C_total_ | 0.003 | 0.001 | -0.000 | 0.012^***^ | 0.009^***^ | 0.009^***^ |
|  | [-0.002,0.007] | [-0.004,0.005] | [-0.005,0.005] | [0.006,0.017] | [0.004,0.015] | [0.003,0.014] |
|  | (0.259) | (0.808) | (0.933) | (0.000) | (0.001) | (0.002) |
|  | (0.002) | (0.002) | (0.002) | (0.003) | (0.003) | (0.003) |
| P_C_ward_ | 0.005 | 0.004 | 0.004 | -0.014^***^ | -0.015^***^ | -0.015^***^ |
|  | [-0.005,0.014] | [-0.005,0.013] | [-0.005,0.014] | [-0.025,-0.004] | [-0.026,-0.005] | [-0.026,-0.004] |
|  | (0.311) | (0.397) | (0.362) | (0.008) | (0.005) | (0.005) |
|  | (0.005) | (0.005) | (0.005) | (0.005) | (0.005) | (0.005) |
| C_total_ | -0.002^***^ | -0.000^**^ | 0.000 | -0.002^***^ | -0.000^**^ | 0.000 |
|  | [-0.003,-0.002] | [-0.001,-0.000] | [-0.000,0.000] | [-0.003,-0.001] | [-0.001,-0.000] | [-0.000,0.000] |
|  | (0.000) | (0.018) | (0.533) | (0.000) | (0.028) | (0.667) |
|  | (0.000) | (0.000) | (0.000) | (0.000) | (0.000) | (0.000) |
| C_PatNum_ | 0.075^***^ | 0.059^***^ | 0.056^***^ | 0.064^***^ | 0.047^***^ | 0.042^**^ |
|  | [0.044,0.106] | [0.028,0.091] | [0.025,0.087] | [0.029,0.099] | [0.012,0.082] | [0.007,0.078] |
|  | (0.000) | (0.000) | (0.000) | (0.000) | (0.008) | (0.018) |
|  | (0.016) | (0.016) | (0.016) | (0.018) | (0.018) | (0.018) |
| day_lag | -0.006^***^ | -0.006^***^ | -0.006^***^ | -0.007^***^ | -0.007^***^ | -0.007^***^ |
|  | [-0.009,-0.003] | [-0.009,-0.003] | [-0.009,-0.003] | [-0.010,-0.005] | [-0.010,-0.005] | [-0.010,-0.005] |
|  | (0.000) | (0.000) | (0.000) | (0.000) | (0.000) | (0.000) |
|  | (0.001) | (0.001) | (0.001) | (0.001) | (0.001) | (0.001) |
| age | 0.000 | 0.000 | 0.000 | 0.001 | 0.001 | 0.001 |
|  | [-0.001,0.002] | [-0.001,0.002] | [-0.001,0.002] | [-0.001,0.002] | [-0.001,0.002] | [-0.001,0.002] |
|  | (0.827) | (0.696) | (0.708) | (0.476) | (0.473) | (0.531) |
|  | (0.001) | (0.001) | (0.001) | (0.001) | (0.001) | (0.001) |
| gender | -0.028 | -0.026 | -0.024 | -0.020 | -0.020 | -0.018 |
|  | [-0.069,0.013] | [-0.067,0.015] | [-0.066,0.017] | [-0.066,0.025] | [-0.066,0.025] | [-0.064,0.028] |
|  | (0.180) | (0.215) | (0.255) | (0.387) | (0.380) | (0.440) |
|  | (0.021) | (0.021) | (0.021) | (0.023) | (0.023) | (0.023) |
| los | -0.003^***^ | -0.003^***^ | -0.003^***^ | -0.002^***^ | -0.002^***^ | -0.002^***^ |
|  | [-0.004,-0.003] | [-0.004,-0.003] | [-0.004,-0.003] | [-0.003,-0.002] | [-0.003,-0.002] | [-0.003,-0.002] |
|  | (0.000) | (0.000) | (0.000) | (0.000) | (0.000) | (0.000) |
|  | (0.000) | (0.000) | (0.000) | (0.000) | (0.000) | (0.000) |
| Diagnosis_count | -0.019 | -0.005 | 0.000 | -0.061 | -0.045 | -0.040 |
|  | [-0.103,0.065] | [-0.090,0.079] | [-0.084,0.085] | [-0.155,0.032] | [-0.139,0.049] | [-0.134,0.055] |
|  | (0.654) | (0.901) | (0.996) | (0.200) | (0.348) | (0.410) |
|  | (0.043) | (0.043) | (0.043) | (0.048) | (0.048) | (0.048) |
| dept | Yes | Yes | Yes | Yes | Yes | Yes |
| spec | Yes | Yes | Yes | Yes | Yes | Yes |
| race | Yes | Yes | Yes | Yes | Yes | Yes |
| day | Yes | Yes | Yes | Yes | Yes | Yes |
| mth | Yes | Yes | Yes | Yes | Yes | Yes |
| constant | -0.192 | -0.607 | -0.941 | -0.164 | -0.509 | -0.819 |
|  | (1.075) | (1.087) | (1.091) | (1.150) | (1.164) | (1.169) |
| *N* | 65540 | 65540 | 65540 | 65555 | 65555 | 65555 |
| pseudo *R*^2^ | 0.129 | 0.136 | 0.141 | 0.132 | 0.139 | 0.145 |
| *AIC* | 80149.239 | 79516.419 | 79071.652 | 68977.402 | 68425.440 | 67969.807 |
| *BIC* | 90094.154 | 89461.334 | 89016.567 | 78940.749 | 78388.787 | 77933.154 |
| Log Likelihood | -38980.620 | -38664.209 | -38441.826 | -33392.701 | -33116.720 | -32888.904 |

**Note:** Coefficients are exponentiated and represent odds ratios. *H_#_* represents habits strength with # as the corresponding habit learning rate. All multivariate models adjusted for the context of the alert, physician's historical exposure to alerts, physician characteristics, patient characteristics, and timing effects. ^***^ indicates *p* < 0.01, ^**^ indicates *p* < 0.05, ^*^ indicates *p* < 0.10.

**Table S4. Random Effects Logistic Regression Models with Dismis and Dismiss1 as Dependent Variables**

| Outcome  Predictor | dismiss | dismiss | dismiss | dismiss1 | dismiss1 | dismiss1 |
| --- | --- | --- | --- | --- | --- | --- |
|  | β coeff.  [95% C.I.]  (P value)  (std.error) | β coeff.  [95% C.I.]  (P value)  (std.error) | β coeff.  [95% C.I.]  (P value)  (std.error) | β coeff.  [95% C.I.]  (P value)  (std.error) | β coeff.  [95% C.I.]  (P value)  (std.error) | β coeff.  [95% C.I.]  (P value)  (std.error) |
| H_0.01_ | 2.512^***^ |  |  | 1.939^***^ |  |  |
|  | [2.104,2.919] |  |  | [1.677,2.201] |  |  |
|  | (0.000) |  |  | (0.000) |  |  |
|  | (0.208) |  |  | (0.134) |  |  |
| H_0.05_ |  | 2.569^***^ |  |  | 1.518^***^ |  |
|  |  | [2.385,2.753] |  |  | [1.376,1.660] |  |
|  |  | (0.000) |  |  | (0.000) |  |
|  |  | (0.094) |  |  | (0.073) |  |
| H_0.1_ |  |  | 2.870^***^ |  |  | 1.717^***^ |
|  |  |  | [2.714,3.026] |  |  | [1.573,1.862] |
|  |  |  | (0.000) |  |  | (0.000) |
|  |  |  | (0.080) |  |  | (0.074) |
| ward | -0.215^***^ | -0.217^***^ | -0.212^***^ | -0.129^***^ | -0.126^***^ | -0.122^***^ |
|  | [-0.292,-0.138] | [-0.295,-0.139] | [-0.291,-0.133] | [-0.182,-0.076] | [-0.179,-0.073] | [-0.175,-0.069] |
|  | (0.000) | (0.000) | (0.000) | (0.000) | (0.000) | (0.000) |
|  | (0.039) | (0.040) | (0.040) | (0.027) | (0.027) | (0.027) |
| P_C_total_ | 0.018^***^ | 0.009 | 0.006 | -0.000 | -0.002 | -0.002 |
|  | [0.005,0.030] | [-0.004,0.022] | [-0.006,0.019] | [-0.006,0.006] | [-0.007,0.004] | [-0.008,0.004] |
|  | (0.006) | (0.158) | (0.314) | (0.960) | (0.598) | (0.489) |
|  | (0.006) | (0.006) | (0.006) | (0.003) | (0.003) | (0.003) |
| P_C_ward_ | -0.036^***^ | -0.031^***^ | -0.030^**^ | 0.009 | 0.010 | 0.010^*^ |
|  | [-0.059,-0.013] | [-0.054,-0.008] | [-0.053,-0.006] | [-0.002,0.021] | [-0.002,0.021] | [-0.002,0.022] |
|  | (0.002) | (0.009) | (0.013) | (0.114) | (0.105) | (0.090) |
|  | (0.012) | (0.012) | (0.012) | (0.006) | (0.006) | (0.006) |
| C_total_ | -0.002^***^ | 0.000 | 0.001^**^ | -0.005^***^ | -0.002^***^ | -0.001^***^ |
|  | [-0.003,-0.001] | [-0.000,0.001] | [0.000,0.002] | [-0.006,-0.004] | [-0.002,-0.001] | [-0.001,-0.000] |
|  | (0.002) | (0.416) | (0.014) | (0.000) | (0.000) | (0.000) |
|  | (0.001) | (0.000) | (0.000) | (0.000) | (0.000) | (0.000) |
| C_PatNum_ | -0.016 | -0.049 | -0.055 | 0.048^**^ | 0.036^*^ | 0.035 |
|  | [-0.082,0.049] | [-0.116,0.017] | [-0.123,0.012] | [0.006,0.090] | [-0.006,0.078] | [-0.007,0.076] |
|  | (0.624) | (0.147) | (0.110) | (0.024) | (0.091) | (0.105) |
|  | (0.034) | (0.034) | (0.035) | (0.021) | (0.021) | (0.021) |
| day_lag | -0.008^***^ | -0.007^***^ | -0.007^***^ | -0.006^**^ | -0.006^**^ | -0.006^**^ |
|  | [-0.012,-0.003] | [-0.012,-0.003] | [-0.012,-0.002] | [-0.011,-0.000] | [-0.011,-0.001] | [-0.011,-0.000] |
|  | (0.002) | (0.002) | (0.005) | (0.037) | (0.030) | (0.039) |
|  | (0.002) | (0.002) | (0.003) | (0.003) | (0.003) | (0.003) |
| C_rank=1 | -0.476^**^ | -0.394^**^ | -0.348^*^ | -0.054 | -0.015 | -0.000 |
|  | [-0.875,-0.077] | [-0.768,-0.021] | [-0.702,0.007] | [-0.218,0.111] | [-0.176,0.146] | [-0.157,0.156] |
|  | (0.019) | (0.039) | (0.055) | (0.524) | (0.855) | (0.997) |
|  | (0.203) | (0.191) | (0.181) | (0.084) | (0.082) | (0.080) |
| C_rank=2 | -0.655^**^ | -0.350 | -0.264 | -0.427^***^ | -0.272^**^ | -0.249^**^ |
|  | [-1.173,-0.137] | [-0.836,0.136] | [-0.725,0.197] | [-0.677,-0.178] | [-0.518,-0.025] | [-0.491,-0.008] |
|  | (0.013) | (0.158) | (0.262) | (0.001) | (0.031) | (0.043) |
|  | (0.264) | (0.248) | (0.235) | (0.127) | (0.126) | (0.123) |
| C_rank=3 | -0.710^***^ | -0.377^*^ | -0.261 | -0.532^***^ | -0.355^***^ | -0.311^***^ |
|  | [-1.188,-0.232] | [-0.824,0.071] | [-0.686,0.164] | [-0.762,-0.302] | [-0.583,-0.127] | [-0.534,-0.089] |
|  | (0.004) | (0.099) | (0.229) | (0.000) | (0.002) | (0.006) |
|  | (0.244) | (0.229) | (0.217) | (0.117) | (0.116) | (0.114) |
| age | -0.001 | -0.002 | -0.002^*^ | 0.001 | 0.001 | 0.001 |
|  | [-0.004,0.002] | [-0.005,0.001] | [-0.005,0.000] | [-0.001,0.003] | [-0.001,0.003] | [-0.001,0.002] |
|  | (0.380) | (0.161) | (0.090) | (0.599) | (0.605) | (0.614) |
|  | (0.001) | (0.001) | (0.001) | (0.001) | (0.001) | (0.001) |
| gender | -0.092^**^ | -0.103^**^ | -0.102^**^ | -0.004 | 0.001 | -0.001 |
|  | [-0.173,-0.012] | [-0.184,-0.021] | [-0.185,-0.019] | [-0.061,0.054] | [-0.056,0.059] | [-0.058,0.057] |
|  | (0.025) | (0.014) | (0.015) | (0.899) | (0.967) | (0.984) |
|  | (0.041) | (0.042) | (0.042) | (0.029) | (0.029) | (0.029) |
| los | 0.001 | 0.001^**^ | 0.001^**^ | -0.003^***^ | -0.003^***^ | -0.003^***^ |
|  | [-0.000,0.002] | [0.000,0.003] | [0.000,0.003] | [-0.004,-0.003] | [-0.004,-0.002] | [-0.004,-0.002] |
|  | (0.108) | (0.037) | (0.028) | (0.000) | (0.000) | (0.000) |
|  | (0.001) | (0.001) | (0.001) | (0.000) | (0.000) | (0.000) |
| Diagnosis_count | 0.040 | 0.065 | 0.057 | -0.119^*^ | -0.111 | -0.105 |
|  | [-0.165,0.245] | [-0.141,0.270] | [-0.150,0.264] | [-0.256,0.018] | [-0.249,0.026] | [-0.242,0.032] |
|  | (0.701) | (0.537) | (0.588) | (0.089) | (0.112) | (0.134) |
|  | (0.105) | (0.105) | (0.106) | (0.070) | (0.070) | (0.070) |
| dept | Yes | Yes | Yes | Yes | Yes | Yes |
| spec | Yes | Yes | Yes | Yes | Yes | Yes |
| race | Yes | Yes | Yes | Yes | Yes | Yes |
| day | Yes | Yes | Yes | Yes | Yes | Yes |
| mth | Yes | Yes | Yes | Yes | Yes | Yes |
| constant | 2.503^**^ | 1.939^*^ | 1.698 | -1.945^*^ | -2.292^**^ | -2.438^**^ |
|  | (1.178) | (1.164) | (1.151) | (1.176) | (1.168) | (1.162) |
| *N* | 66046 | 66046 | 66046 | 66046 | 66046 | 66046 |
| *AIC* | 25658.800 | 25061.992 | 24511.515 | 45798.986 | 45557.197 | 45426.195 |
| *BIC* | 26659.592 | 26062.784 | 25512.306 | 46799.777 | 46557.988 | 46426.987 |
| Log Likelihood | -12719.400 | -12420.996 | -12145.757 | -22789.493 | -22668.598 | -22603.098 |

**Note:** Coefficients are exponentiated and represent odds ratios. *H_#_* represents habits strength with # as the corresponding habit learning rate. All multivariate models adjusted for the context of the alert, physician's historical exposure to alerts, physician characteristics, patient characteristics, and timing effects. ^***^ indicates *p* < 0.01, ^**^ indicates *p* < 0.05, ^*^ indicates *p* < 0.10. C_rank = 0 if physician level is an intern (first year post-medical school), C_rank = 1 if physician level is a resident (2 to 6 years of work experience), C_rank = 2 if physician level is a fellow (completed residency and in specialist training), and C_rank = 3 if physician level is an attending physician (specialists). We use the intern level as the reference group.

**Table S5. Random Effects Logistic Regression Models with Dismiss2 and Dismiss3 as Dependent Variables**

| Outcome  Predictor | dismiss2 | dismiss2 | dismiss2 | dismiss3 | dismiss3 | dismiss3 |
| --- | --- | --- | --- | --- | --- | --- |
|  | β coeff.  [95% C.I.]  (P value)  (std.error) | β coeff.  [95% C.I.]  (P value)  (std.error) | β coeff.  [95% C.I.]  (P value)  (std.error) | β coeff.  [95% C.I.]  (P value)  (std.error) | β coeff.  [95% C.I.]  (P value)  (std.error) | β coeff.  [95% C.I.]  (P value)  (std.error) |
| H_0.01_ | 1.456^***^ |  |  | 1.470^***^ |  |  |
|  | [1.284,1.629] |  |  | [1.279,1.661] |  |  |
|  | (0.000) |  |  | (0.000) |  |  |
|  | (0.088) |  |  | (0.097) |  |  |
| H_0.05_ |  | 1.532^***^ |  |  | 1.529^***^ |  |
|  |  | [1.439,1.626] |  |  | [1.430,1.627] |  |
|  |  | (0.000) |  |  | (0.000) |  |
|  |  | (0.048) |  |  | (0.050) |  |
| H_0.1_ |  |  | 1.827^***^ |  |  | 1.828^***^ |
|  |  |  | [1.736,1.919] |  |  | [1.736,1.920] |
|  |  |  | (0.000) |  |  | (0.000) |
|  |  |  | (0.048) |  |  | (0.050) |
| ward | -0.199^***^ | -0.198^***^ | -0.194^***^ | -0.209^***^ | -0.206^***^ | -0.202^***^ |
|  | [-0.237,-0.162] | [-0.235,-0.160] | [-0.232,-0.156] | [-0.250,-0.168] | [-0.248,-0.165] | [-0.244,-0.161] |
|  | (0.000) | (0.000) | (0.000) | (0.000) | (0.000) | (0.000) |
|  | (0.019) | (0.019) | (0.019) | (0.021) | (0.021) | (0.021) |
| P_C_total_ | 0.002 | 0.000 | -0.000 | 0.011^***^ | 0.009^***^ | 0.008^***^ |
|  | [-0.002,0.007] | [-0.004,0.005] | [-0.005,0.004] | [0.006,0.017] | [0.004,0.015] | [0.003,0.014] |
|  | (0.308) | (0.858) | (0.880) | (0.000) | (0.001) | (0.002) |
|  | (0.002) | (0.002) | (0.002) | (0.003) | (0.003) | (0.003) |
| P_C_ward_ | 0.006 | 0.005 | 0.006 | -0.013^**^ | -0.014^***^ | -0.014^***^ |
|  | [-0.003,0.015] | [-0.004,0.015] | [-0.003,0.015] | [-0.024,-0.003] | [-0.024,-0.003] | [-0.024,-0.003] |
|  | (0.200) | (0.245) | (0.222) | (0.013) | (0.009) | (0.009) |
|  | (0.005) | (0.005) | (0.005) | (0.005) | (0.005) | (0.005) |
| C_total_ | -0.003^***^ | -0.001^***^ | -0.000^***^ | -0.003^***^ | -0.001^***^ | -0.001^***^ |
|  | [-0.003,-0.002] | [-0.001,-0.001] | [-0.001,-0.000] | [-0.003,-0.002] | [-0.002,-0.001] | [-0.001,-0.000] |
|  | (0.000) | (0.000) | (0.010) | (0.000) | (0.000) | (0.001) |
|  | (0.000) | (0.000) | (0.000) | (0.000) | (0.000) | (0.000) |
| C_PatNum_ | 0.073^***^ | 0.056^***^ | 0.052^***^ | 0.060^***^ | 0.041^**^ | 0.035^*^ |
|  | [0.043,0.104] | [0.025,0.087] | [0.021,0.083] | [0.025,0.095] | [0.006,0.076] | [-0.000,0.069] |
|  | (0.000) | (0.000) | (0.001) | (0.001) | (0.021) | (0.052) |
|  | (0.016) | (0.016) | (0.016) | (0.018) | (0.018) | (0.018) |
| day_lag | -0.007^***^ | -0.007^***^ | -0.007^***^ | -0.008^***^ | -0.008^***^ | -0.008^***^ |
|  | [-0.010,-0.004] | [-0.010,-0.004] | [-0.010,-0.004] | [-0.010,-0.005] | [-0.010,-0.005] | [-0.010,-0.005] |
|  | (0.000) | (0.000) | (0.000) | (0.000) | (0.000) | (0.000) |
|  | (0.001) | (0.001) | (0.001) | (0.001) | (0.001) | (0.001) |
| C_rank=1 | -0.113 | -0.057 | -0.027 | -0.034 | 0.018 | 0.048 |
|  | [-0.266,0.040] | [-0.203,0.088] | [-0.166,0.112] | [-0.194,0.126] | [-0.132,0.167] | [-0.093,0.188] |
|  | (0.148) | (0.438) | (0.704) | (0.674) | (0.816) | (0.504) |
|  | (0.078) | (0.074) | (0.071) | (0.082) | (0.076) | (0.072) |
| C_rank=2 | -0.432^***^ | -0.226^**^ | -0.168^*^ | -0.272^**^ | -0.069 | -0.005 |
|  | [-0.639,-0.225] | [-0.424,-0.027] | [-0.360,0.023] | [-0.487,-0.057] | [-0.272,0.133] | [-0.197,0.187] |
|  | (0.000) | (0.026) | (0.084) | (0.013) | (0.502) | (0.961) |
|  | (0.106) | (0.101) | (0.098) | (0.110) | (0.103) | (0.098) |
| C_rank=3 | -0.589^***^ | -0.365^***^ | -0.287^***^ | -0.363^***^ | -0.141 | -0.056 |
|  | [-0.781,-0.396] | [-0.550,-0.181] | [-0.464,-0.109] | [-0.562,-0.164] | [-0.328,0.046] | [-0.233,0.122] |
|  | (0.000) | (0.000) | (0.002) | (0.000) | (0.140) | (0.537) |
|  | (0.098) | (0.094) | (0.091) | (0.102) | (0.096) | (0.091) |
| age | 0.000 | 0.000 | 0.000 | 0.000 | 0.000 | 0.000 |
|  | [-0.001,0.001] | [-0.001,0.002] | [-0.001,0.002] | [-0.001,0.002] | [-0.001,0.002] | [-0.001,0.002] |
|  | (0.880) | (0.805) | (0.851) | (0.669) | (0.727) | (0.840) |
|  | (0.001) | (0.001) | (0.001) | (0.001) | (0.001) | (0.001) |
| gender | -0.027 | -0.025 | -0.023 | -0.019 | -0.018 | -0.015 |
|  | [-0.068,0.013] | [-0.065,0.016] | [-0.063,0.018] | [-0.063,0.026] | [-0.063,0.027] | [-0.060,0.030] |
|  | (0.190) | (0.237) | (0.280) | (0.417) | (0.430) | (0.505) |
|  | (0.021) | (0.021) | (0.021) | (0.023) | (0.023) | (0.023) |
| los | -0.003^***^ | -0.003^***^ | -0.003^***^ | -0.002^***^ | -0.002^***^ | -0.002^***^ |
|  | [-0.004,-0.003] | [-0.004,-0.003] | [-0.004,-0.003] | [-0.003,-0.002] | [-0.003,-0.002] | [-0.002,-0.001] |
|  | (0.000) | (0.000) | (0.000) | (0.000) | (0.000) | (0.000) |
|  | (0.000) | (0.000) | (0.000) | (0.000) | (0.000) | (0.000) |
| Diagnosis_count | -0.020 | -0.005 | 0.001 | -0.060 | -0.041 | -0.035 |
|  | [-0.103,0.063] | [-0.088,0.079] | [-0.082,0.084] | [-0.152,0.032] | [-0.134,0.051] | [-0.127,0.057] |
|  | (0.636) | (0.914) | (0.978) | (0.202) | (0.381) | (0.457) |
|  | (0.042) | (0.042) | (0.042) | (0.047) | (0.047) | (0.047) |
| dept | Yes | Yes | Yes | Yes | Yes | Yes |
| spec | Yes | Yes | Yes | Yes | Yes | Yes |
| race | Yes | Yes | Yes | Yes | Yes | Yes |
| day | Yes | Yes | Yes | Yes | Yes | Yes |
| mth | Yes | Yes | Yes | Yes | Yes | Yes |
| constant | -0.309 | -0.721 | -0.913 | -0.042 | -0.424 | -0.607 |
|  | (0.819) | (0.813) | (0.807) | (0.805) | (0.793) | (0.783) |
| *N* | 66046 | 66046 | 66046 | 66046 | 66046 | 66046 |
| *AIC* | 81281.397 | 80530.063 | 79987.844 | 70087.421 | 69415.341 | 68847.030 |
| *BIC* | 82282.189 | 81530.855 | 80988.636 | 71088.213 | 70416.133 | 69847.821 |
| Log Likelihood | -40530.698 | -40155.032 | -39883.922 | -34933.711 | -34597.671 | -34313.515 |

**Note:** Coefficients are exponentiated and represent odds ratios. *H_#_* represents habits strength with # as the corresponding habit learning rate. All multivariate models adjusted for the context of the alert, physician's historical exposure to alerts, physician characteristics, patient characteristics, and timing effects. ^***^ indicates *p* < 0.01, ^**^ indicates *p* < 0.05, ^*^ indicates *p* < 0.10. C_rank = 0 if physician level is an intern (first year post-medical school), C_rank = 1 if physician level is a resident (2 to 6 years of work experience), C_rank = 2 if physician level is a fellow (completed residency and in specialist training), and C_rank = 3 if physician level is an attending physician (specialists). We use the intern level as the reference group.

**Table S6. Regular Logistic Regression Models with Dismiss and Dismiss1 as Dependent Variables**

| Outcome  Predictor | dismiss | dismiss | dismiss | dismiss1 | dismiss1 | dismiss1 |
| --- | --- | --- | --- | --- | --- | --- |
|  | β coeff.  [95% C.I.]  (P value)  (std.error) | β coeff.  [95% C.I.]  (P value)  (std.error) | β coeff.  [95% C.I.]  (P value)  (std.error) | β coeff.  [95% C.I.]  (P value)  (std.error) | β coeff.  [95% C.I.]  (P value)  (std.error) | β coeff.  [95% C.I.]  (P value)  (std.error) |
|  |  |  |  |  |  |  |
| H_0.01_ | 6.966^***^ |  |  | 2.514^***^ |  |  |
|  | [6.717,7.215] |  |  | [2.315,2.713] |  |  |
|  | (0.000) |  |  | (0.000) |  |  |
|  | (0.127) |  |  | (0.102) |  |  |
| H_0.05_ |  | 4.122^***^ |  |  | 1.724^***^ |  |
|  |  | [4.000,4.245] |  |  | [1.614,1.834] |  |
|  |  | (0.000) |  |  | (0.000) |  |
|  |  | (0.062) |  |  | (0.056) |  |
| H_0.1_ |  |  | 4.160^***^ |  |  | 1.948^***^ |
|  |  |  | [4.051,4.270] |  |  | [1.831,2.065] |
|  |  |  | (0.000) |  |  | (0.000) |
|  |  |  | (0.056) |  |  | (0.060) |
| ward | -0.200^***^ | -0.202^***^ | -0.197^***^ | -0.125^***^ | -0.121^***^ | -0.116^***^ |
|  | [-0.266,-0.134] | [-0.269,-0.135] | [-0.266,-0.128] | [-0.176,-0.074] | [-0.172,-0.070] | [-0.167,-0.065] |
|  | (0.000) | (0.000) | (0.000) | (0.000) | (0.000) | (0.000) |
|  | (0.034) | (0.034) | (0.035) | (0.026) | (0.026) | (0.026) |
| P_C_total_ | 0.011^**^ | 0.007 | 0.008^*^ | 0.002 | 0.001 | 0.000 |
|  | [0.001,0.020] | [-0.002,0.016] | [-0.001,0.017] | [-0.003,0.007] | [-0.005,0.006] | [-0.005,0.005] |
|  | (0.027) | (0.139) | (0.095) | (0.496) | (0.816) | (0.936) |
|  | (0.005) | (0.005) | (0.005) | (0.003) | (0.003) | (0.003) |
| P_C_ward_ | -0.030^***^ | -0.025^***^ | -0.027^***^ | 0.010^*^ | 0.011^**^ | 0.011^**^ |
|  | [-0.048,-0.012] | [-0.042,-0.007] | [-0.045,-0.009] | [-0.000,0.021] | [0.000,0.021] | [0.000,0.021] |
|  | (0.001) | (0.006) | (0.003) | (0.052) | (0.043) | (0.040) |
|  | (0.009) | (0.009) | (0.009) | (0.005) | (0.005) | (0.005) |
| C_total_ | -0.012^***^ | -0.005^***^ | -0.003^***^ | -0.006^***^ | -0.002^***^ | -0.001^***^ |
|  | [-0.013,-0.012] | [-0.005,-0.004] | [-0.003,-0.003] | [-0.007,-0.005] | [-0.002,-0.001] | [-0.001,-0.000] |
|  | (0.000) | (0.000) | (0.000) | (0.000) | (0.000) | (0.000) |
|  | (0.000) | (0.000) | (0.000) | (0.000) | (0.000) | (0.000) |
| C_PatNum_ | -0.160^***^ | -0.178^***^ | -0.176^***^ | 0.027 | 0.020 | 0.021 |
|  | [-0.216,-0.104] | [-0.234,-0.122] | [-0.233,-0.119] | [-0.012,0.066] | [-0.019,0.059] | [-0.018,0.060] |
|  | (0.000) | (0.000) | (0.000) | (0.172) | (0.322) | (0.294) |
|  | (0.029) | (0.029) | (0.029) | (0.020) | (0.020) | (0.020) |
| day_lag | -0.010^***^ | -0.011^***^ | -0.011^***^ | -0.011^***^ | -0.010^***^ | -0.010^***^ |
|  | [-0.014,-0.007] | [-0.014,-0.007] | [-0.014,-0.007] | [-0.016,-0.005] | [-0.015,-0.005] | [-0.015,-0.005] |
|  | (0.000) | (0.000) | (0.000) | (0.000) | (0.000) | (0.000) |
|  | (0.002) | (0.002) | (0.002) | (0.003) | (0.003) | (0.003) |
| C_rank=1 | -0.071 | -0.104^**^ | -0.103^**^ | 0.026 | 0.028 | 0.030 |
|  | [-0.158,0.017] | [-0.194,-0.014] | [-0.195,-0.010] | [-0.032,0.084] | [-0.030,0.086] | [-0.029,0.088] |
|  | (0.114) | (0.023) | (0.030) | (0.382) | (0.347) | (0.319) |
|  | (0.045) | (0.046) | (0.047) | (0.030) | (0.030) | (0.030) |
| C_rank=2 | 0.150^**^ | 0.230^***^ | 0.205^***^ | -0.202^***^ | -0.130^*^ | -0.150^**^ |
|  | [0.015,0.286] | [0.089,0.370] | [0.060,0.351] | [-0.331,-0.072] | [-0.260,0.000] | [-0.280,-0.020] |
|  | (0.030) | (0.001) | (0.006) | (0.002) | (0.050) | (0.023) |
|  | (0.069) | (0.072) | (0.074) | (0.066) | (0.066) | (0.066) |
| C_rank=3 | -0.212^***^ | -0.120^*^ | -0.112^*^ | -0.234^***^ | -0.142^**^ | -0.140^**^ |
|  | [-0.329,-0.095] | [-0.241,0.002] | [-0.238,0.014] | [-0.346,-0.122] | [-0.255,-0.029] | [-0.253,-0.027] |
|  | (0.000) | (0.054) | (0.081) | (0.000) | (0.014) | (0.015) |
|  | (0.060) | (0.062) | (0.064) | (0.057) | (0.058) | (0.058) |
| age | -0.003^**^ | -0.003^***^ | -0.004^***^ | -0.000 | -0.000 | -0.000 |
|  | [-0.005,-0.000] | [-0.006,-0.001] | [-0.006,-0.002] | [-0.002,0.002] | [-0.002,0.001] | [-0.002,0.001] |
|  | (0.031) | (0.005) | (0.001) | (0.774) | (0.625) | (0.663) |
|  | (0.001) | (0.001) | (0.001) | (0.001) | (0.001) | (0.001) |
| gender | -0.057^*^ | -0.063^*^ | -0.064^*^ | -0.010 | -0.005 | -0.005 |
|  | [-0.123,0.009] | [-0.131,0.004] | [-0.133,0.006] | [-0.063,0.042] | [-0.058,0.048] | [-0.058,0.048] |
|  | (0.091) | (0.067) | (0.073) | (0.701) | (0.847) | (0.846) |
|  | (0.034) | (0.035) | (0.036) | (0.027) | (0.027) | (0.027) |
| los | 0.002^***^ | 0.002^***^ | 0.002^***^ | -0.004^***^ | -0.003^***^ | -0.003^***^ |
|  | [0.001,0.003] | [0.001,0.003] | [0.001,0.003] | [-0.004,-0.003] | [-0.004,-0.003] | [-0.004,-0.003] |
|  | (0.002) | (0.000) | (0.000) | (0.000) | (0.000) | (0.000) |
|  | (0.001) | (0.001) | (0.001) | (0.000) | (0.000) | (0.000) |
| Diagnosis_count | 0.030 | 0.047 | 0.045 | -0.176^***^ | -0.155^**^ | -0.141^**^ |
|  | [-0.123,0.182] | [-0.105,0.199] | [-0.110,0.200] | [-0.302,-0.051] | [-0.280,-0.029] | [-0.267,-0.015] |
|  | (0.703) | (0.546) | (0.573) | (0.006) | (0.015) | (0.028) |
|  | (0.078) | (0.077) | (0.079) | (0.064) | (0.064) | (0.064) |
| dept | Yes | Yes | Yes | Yes | Yes | Yes |
| spec | Yes | Yes | Yes | Yes | Yes | Yes |
| race | Yes | Yes | Yes | Yes | Yes | Yes |
| day | Yes | Yes | Yes | Yes | Yes | Yes |
| mth | Yes | Yes | Yes | Yes | Yes | Yes |
| constant | 1.499^*^ | 1.154 | 0.945 | -1.956^*^ | -2.213^**^ | -2.353^**^ |
|  | (0.827) | (0.830) | (0.835) | (1.074) | (1.074) | (1.075) |
| *N* | 66046 | 66046 | 66046 | 66046 | 66046 | 66046 |
| pseudo *R*^2^ | 0.219 | 0.254 | 0.290 | 0.087 | 0.093 | 0.098 |
| *AIC* | 30956.247 | 29570.211 | 28156.467 | 47402.807 | 47047.558 | 46815.121 |
| *BIC* | 31947.941 | 30561.905 | 29148.161 | 48394.500 | 48039.252 | 47806.814 |
| Log Likelihood | -15369.124 | -14676.106 | -13969.233 | -23592.403 | -23414.779 | -23298.560 |

**Note:** Coefficients are exponentiated and represent odds ratios. *H_#_* represents habits strength with # as the corresponding habit learning rate. All multivariate models adjusted for the context of the alert, physician's historical exposure to alerts, physician characteristics, patient characteristics, and timing effects. ^***^ indicates *p* < 0.01, ^**^ indicates *p* < 0.05, ^*^ indicates *p* < 0.10. C_rank = 0 if physician level is an intern (first year post-medical school), C_rank = 1 if physician level is a resident (2 to 6 years of work experience), C_rank = 2 if physician level is a fellow (completed residency and in specialist training), and C_rank = 3 if physician level is an attending physician (specialists). We use the intern level as the reference group.

**Table S7. Regular Logistic Regression Models with Dismiss2 and Dismiss3 as Dependent Variables**

| Outcome  Predictor | dismiss2 | dismiss2 | dismiss2 | dismiss3 | dismiss3 | dismiss3 |
| --- | --- | --- | --- | --- | --- | --- |
|  | β coeff.  [95% C.I.]  (P value)  (std.error) | β coeff.  [95% C.I.]  (P value)  (std.error) | β coeff.  [95% C.I.]  (P value)  (std.error) | β coeff.  [95% C.I.]  (P value)  (std.error) | β coeff.  [95% C.I.]  (P value)  (std.error) | β coeff.  [95% C.I.]  (P value)  (std.error) |
|  |  |  |  |  |  |  |
| H_0.01_ | 2.574^***^ |  |  | 2.918^***^ |  |  |
|  | [2.455,2.694] |  |  | [2.791,3.046] |  |  |
|  | (0.000) |  |  | (0.000) |  |  |
|  | (0.061) |  |  | (0.065) |  |  |
| H_0.05_ |  | 1.928^***^ |  |  | 2.101^***^ |  |
|  |  | [1.858,1.999] |  |  | [2.028,2.175] |  |
|  |  | (0.000) |  |  | (0.000) |  |
|  |  | (0.036) |  |  | (0.037) |  |
| H_0.1_ |  |  | 2.210^***^ |  |  | 2.351^***^ |
|  |  |  | [2.138,2.281] |  |  | [2.280,2.423] |
|  |  |  | (0.000) |  |  | (0.000) |
|  |  |  | (0.037) |  |  | (0.037) |
| ward | -0.202^***^ | -0.197^***^ | -0.192^***^ | -0.214^***^ | -0.209^***^ | -0.203^***^ |
|  | [-0.237,-0.167] | [-0.233,-0.162] | [-0.227,-0.156] | [-0.253,-0.176] | [-0.248,-0.170] | [-0.243,-0.164] |
|  | (0.000) | (0.000) | (0.000) | (0.000) | (0.000) | (0.000) |
|  | (0.018) | (0.018) | (0.018) | (0.020) | (0.020) | (0.020) |
| P_C_total_ | -0.002 | -0.003 | -0.004^*^ | 0.006^**^ | 0.005^**^ | 0.005^**^ |
|  | [-0.006,0.002] | [-0.007,0.001] | [-0.008,0.000] | [0.001,0.011] | [0.000,0.010] | [0.000,0.009] |
|  | (0.306) | (0.124) | (0.081) | (0.012) | (0.034) | (0.043) |
|  | (0.002) | (0.002) | (0.002) | (0.002) | (0.002) | (0.002) |
| P_C_ward_ | 0.017^***^ | 0.016^***^ | 0.015^***^ | -0.003 | -0.004 | -0.004 |
|  | [0.008,0.025] | [0.007,0.024] | [0.007,0.023] | [-0.012,0.007] | [-0.013,0.005] | [-0.013,0.005] |
|  | (0.000) | (0.000) | (0.000) | (0.587) | (0.419) | (0.351) |
|  | (0.004) | (0.004) | (0.004) | (0.005) | (0.005) | (0.005) |
| C_total_ | -0.006^***^ | -0.002^***^ | -0.001^***^ | -0.006^***^ | -0.002^***^ | -0.002^***^ |
|  | [-0.006,-0.005] | [-0.002,-0.002] | [-0.001,-0.001] | [-0.007,-0.006] | [-0.003,-0.002] | [-0.002,-0.001] |
|  | (0.000) | (0.000) | (0.000) | (0.000) | (0.000) | (0.000) |
|  | (0.000) | (0.000) | (0.000) | (0.000) | (0.000) | (0.000) |
| C_PatNum_ | 0.042^***^ | 0.030^**^ | 0.029^**^ | 0.011 | -0.003 | -0.004 |
|  | [0.014,0.070] | [0.001,0.058] | [0.000,0.057] | [-0.021,0.043] | [-0.035,0.029] | [-0.036,0.027] |
|  | (0.004) | (0.040) | (0.047) | (0.497) | (0.862) | (0.783) |
|  | (0.014) | (0.014) | (0.015) | (0.016) | (0.016) | (0.016) |
| day_lag | -0.008^***^ | -0.008^***^ | -0.008^***^ | -0.009^***^ | -0.009^***^ | -0.008^***^ |
|  | [-0.011,-0.006] | [-0.011,-0.005] | [-0.010,-0.005] | [-0.012,-0.006] | [-0.011,-0.006] | [-0.011,-0.006] |
|  | (0.000) | (0.000) | (0.000) | (0.000) | (0.000) | (0.000) |
|  | (0.001) | (0.001) | (0.001) | (0.001) | (0.001) | (0.001) |
| C_rank=1 | 0.069^***^ | 0.066^***^ | 0.072^***^ | 0.102^***^ | 0.097^***^ | 0.104^***^ |
|  | [0.028,0.110] | [0.025,0.107] | [0.030,0.113] | [0.056,0.148] | [0.050,0.143] | [0.057,0.151] |
|  | (0.001) | (0.002) | (0.001) | (0.000) | (0.000) | (0.000) |
|  | (0.021) | (0.021) | (0.021) | (0.023) | (0.024) | (0.024) |
| C_rank=2 | -0.079^**^ | -0.021 | -0.035 | 0.087^**^ | 0.148^***^ | 0.139^***^ |
|  | [-0.156,-0.003] | [-0.099,0.057] | [-0.114,0.044] | [0.005,0.170] | [0.065,0.232] | [0.054,0.225] |
|  | (0.042) | (0.603) | (0.382) | (0.037) | (0.001) | (0.001) |
|  | (0.039) | (0.040) | (0.040) | (0.042) | (0.043) | (0.043) |
| C_rank=3 | -0.157^***^ | -0.090^***^ | -0.081^**^ | -0.029 | 0.039 | 0.056 |
|  | [-0.224,-0.090] | [-0.158,-0.022] | [-0.150,-0.013] | [-0.101,0.042] | [-0.034,0.113] | [-0.018,0.131] |
|  | (0.000) | (0.009) | (0.020) | (0.423) | (0.291) | (0.138) |
|  | (0.034) | (0.035) | (0.035) | (0.037) | (0.037) | (0.038) |
| age | 0.000 | 0.000 | 0.000 | 0.000 | 0.000 | 0.000 |
|  | [-0.001,0.001] | [-0.001,0.001] | [-0.001,0.001] | [-0.001,0.002] | [-0.001,0.002] | [-0.001,0.002] |
|  | (0.906) | (0.956) | (0.984) | (0.564) | (0.631) | (0.701) |
|  | (0.001) | (0.001) | (0.001) | (0.001) | (0.001) | (0.001) |
| gender | -0.031^*^ | -0.026 | -0.025 | -0.021 | -0.017 | -0.015 |
|  | [-0.067,0.005] | [-0.063,0.011] | [-0.062,0.012] | [-0.061,0.019] | [-0.057,0.024] | [-0.056,0.026] |
|  | (0.096) | (0.167) | (0.193) | (0.310) | (0.413) | (0.477) |
|  | (0.019) | (0.019) | (0.019) | (0.020) | (0.021) | (0.021) |
| los | -0.003^***^ | -0.003^***^ | -0.003^***^ | -0.002^***^ | -0.001^***^ | -0.001^***^ |
|  | [-0.003,-0.002] | [-0.003,-0.002] | [-0.003,-0.002] | [-0.002,-0.001] | [-0.002,-0.001] | [-0.002,-0.001] |
|  | (0.000) | (0.000) | (0.000) | (0.000) | (0.000) | (0.000) |
|  | (0.000) | (0.000) | (0.000) | (0.000) | (0.000) | (0.000) |
| Diagnosis_count | -0.067^*^ | -0.047 | -0.038 | -0.079^*^ | -0.059 | -0.051 |
|  | [-0.139,0.006] | [-0.120,0.026] | [-0.111,0.035] | [-0.159,0.001] | [-0.139,0.021] | [-0.132,0.030] |
|  | (0.072) | (0.206) | (0.310) | (0.054) | (0.150) | (0.221) |
|  | (0.037) | (0.037) | (0.037) | (0.041) | (0.041) | (0.041) |
| dept | Yes | Yes | Yes | Yes | Yes | Yes |
| spec | Yes | Yes | Yes | Yes | Yes | Yes |
| race | Yes | Yes | Yes | Yes | Yes | Yes |
| day | Yes | Yes | Yes | Yes | Yes | Yes |
| mth | Yes | Yes | Yes | Yes | Yes | Yes |
| constant | -0.593 | -0.853 | -1.031 | -0.157 | -0.421 | -0.601 |
|  | (0.718) | (0.718) | (0.719) | (0.683) | (0.684) | (0.686) |
| *N* | 66046 | 66046 | 66046 | 66046 | 66046 | 66046 |
| pseudo *R*^2^ | 0.052 | 0.065 | 0.076 | 0.049 | 0.065 | 0.079 |
| *AIC* | 85847.121 | 84654.336 | 83700.627 | 74081.577 | 72884.275 | 71801.984 |
| *BIC* | 86838.815 | 85646.030 | 84692.321 | 75073.271 | 73875.969 | 72793.678 |
| Log Likelihood | -42814.561 | -42218.168 | -41741.314 | -36931.789 | -36333.138 | -35791.992 |

**Note:** Coefficients are exponentiated and represent odds ratios. *H_#_* represents habits strength with # as the corresponding habit learning rate. All multivariate models adjusted for the context of the alert, physician's historical exposure to alerts, physician characteristics, patient characteristics, and timing effects. ^***^ indicates *p* < 0.01, ^**^ indicates *p* < 0.05, ^*^ indicates *p* < 0.10. C_rank = 0 if physician level is an intern (first year post-medical school), C_rank = 1 if physician level is a resident (2 to 6 years of work experience), C_rank = 2 if physician level is a fellow (completed residency and in specialist training), and C_rank = 3 if physician level is an attending physician (specialists). We use the intern level as the reference group.

**Table S8. Fixed Effects Logistic Regression Models for Intern Subsample**

| Outcome  Predictor | dismiss | dismiss1 | dismiss2 | dismiss3 |
| --- | --- | --- | --- | --- |
|  | β coeff.  [95% C.I.]  (P value)  (std.error) | β coeff.  [95% C.I.]  (P value)  (std.error) | β coeff.  [95% C.I.]  (P value)  (std.error) | β coeff.  [95% C.I.]  (P value)  (std.error) |
|  |  |  |  |  |
| H_0.05_ | 3.235^***^ | 1.656^***^ | 1.476^***^ | 1.560^***^ |
|  | [2.785,3.685] | [1.368,1.944] | [1.288,1.664] | [1.364,1.756] |
|  | (0.000) | (0.000) | (0.000) | (0.000) |
| ward | (0.230) | (0.147) | (0.096) | (0.100) |
|  | -0.371^***^ | -0.112^**^ | -0.242^***^ | -0.284^***^ |
|  | [-0.568,-0.175] | [-0.214,-0.010] | [-0.316,-0.169] | [-0.366,-0.202] |
|  | (0.000) | (0.032) | (0.000) | (0.000) |
| P_C_total_ | (0.100) | (0.052) | (0.038) | (0.042) |
|  | -0.008 | 0.006 | -0.004 | -0.002 |
|  | [-0.033,0.016] | [-0.004,0.016] | [-0.012,0.004] | [-0.011,0.007] |
|  | (0.510) | (0.273) | (0.280) | (0.608) |
| P_C_ward_ | (0.013) | (0.005) | (0.165) | (0.185) |
|  | 0.016 | -0.007 | 0.017^**^ | 0.007 |
|  | [-0.035,0.068] | [-0.028,0.015] | [0.000,0.033] | [-0.011,0.026] |
|  | (0.530) | (0.544) | (0.045) | (0.437) |
| C_total_ | (0.026) | (0.011) | (0.008) | (0.009) |
|  | -0.004^***^ | -0.001 | -0.001^**^ | -0.002^***^ |
|  | [-0.006,-0.002] | [-0.002,0.000] | [-0.001,-0.000] | [-0.002,-0.001] |
|  | (0.000) | (0.101) | (0.037) | (0.000) |
| C_PatNum_ | (0.001) | (0.000) | (0.000) | (0.000) |
|  | -0.181^**^ | 0.042 | 0.022 | -0.042 |
|  | [-0.336,-0.027] | [-0.027,0.111] | [-0.034,0.077] | [-0.104,0.019] |
|  | (0.021) | (0.234) | (0.443) | (0.179) |
|  | (0.079) | (0.035) | (0.028) | (0.032) |
| day_lag | -0.035^***^ | -0.022^***^ | -0.015^***^ | -0.009^***^ |
|  | [-0.059,-0.012] | [-0.035,-0.009] | [-0.023,-0.008] | [-0.016,-0.003] |
|  | (0.003) | (0.001) | (0.000) | (0.006) |
|  | (0.012) | (0.007) | (0.004) | (0.003) |
| age | -0.010^**^ | 0.002 | 0.001 | 0.003^*^ |
|  | [-0.018,-0.002] | [-0.003,0.006] | [-0.002,0.004] | [-0.000,0.006] |
|  | (0.012) | (0.465) | (0.511) | (0.058) |
|  | (0.004) | (0.002) | (0.002) | (0.002) |
| gender | -0.154 | -0.071 | -0.048 | -0.028 |
|  | [-0.388,0.080] | [-0.187,0.046] | [-0.133,0.036] | [-0.122,0.065] |
|  | (0.197) | (0.233) | (0.262) | (0.552) |
|  | (0.119) | (0.059) | (0.043) | (0.048) |
| loc | 0.002 | -0.006^***^ | -0.005^***^ | -0.002^***^ |
|  | [-0.003,0.007] | [-0.008,-0.003] | [-0.006,-0.003] | [-0.003,-0.001] |
|  | (0.461) | (0.000) | (0.000) | (0.001) |
|  | (0.002) | (0.001) | (0.001) | (0.001) |
| Diagnosis_count | 0.328 | -0.108 | 0.101 | 0.059 |
|  | [-0.247,0.903] | [-0.358,0.142] | [-0.062,0.265] | [-0.125,0.243] |
|  | (0.264) | (0.398) | (0.225) | (0.531) |
|  | (0.294) | (0.128) | (0.084) | (0.094) |
| dept | Yes | Yes | Yes | Yes |
| spec | Yes | Yes | Yes | Yes |
| race | Yes | Yes | Yes | Yes |
| day | Yes | Yes | Yes | Yes |
| mth | Yes | Yes | Yes | Yes |
| constant | 2.488^***^ | -2.902^***^ | 0.216 | 0.407 |
|  | [0.896,4.080] | [-3.801,-2.003] | [-0.410,0.842] | [-0.264,1.078] |
| *N* | 16134 | 16319 | 16347 | 16314 |
| *AIC* | 4133.155 | 11898.736 | 20188.067 | 17181.164 |
| *BIC* | 4802.070 | 12630.244 | 20965.949 | 17928.043 |
| Log Likelihood | -1979.577 | -5854.368 | -9993.033 | -8493.582 |

**Note:** We use the subsample of physicians with the level of intern for this analysis. Coefficients are exponentiated and represent odds ratios. *H_#_* represents habits strength with # as the corresponding habit learning rate. All multivariate models adjusted for the context of the alert, physician's historical exposure to alerts, physician characteristics, patient characteristics, and timing effects. ^***^ indicates *p* < 0.01, ^**^ indicates *p* < 0.05, ^*^ indicates *p* < 0.10.

**Table S9. Fixed Effects Logistic Regression Models for Resident Subsample**

| Outcome  Predictor | dismiss | dismiss1 | dismiss2 | dismiss3 |
| --- | --- | --- | --- | --- |
|  | β coeff.  [95% C.I.]  (P value)  (std.error) | β coeff.  [95% C.I.]  (P value)  (std.error) | β coeff.  [95% C.I.]  (P value)  (std.error) | β coeff.  [95% C.I.]  (P value)  (std.error) |
|  |  |  |  |  |
| H_0.05_ | 2.604^***^ | 1.354^***^ | 1.496^***^ | 1.486^***^ |
|  | [2.374,2.835] | [1.174,1.535] | [1.374,1.618] | [1.357,1.614] |
|  | (0.000) | (0.000) | (0.000) | (0.000) |
|  | (0.118) | (0.092) | (0.062) | (0.066) |
| ward | -0.206^***^ | -0.122^***^ | -0.197^***^ | -0.183^***^ |
|  | [-0.304,-0.108] | [-0.190,-0.055] | [-0.245,-0.148] | [-0.237,-0.128] |
|  | (0.000) | (0.000) | (0.000) | (0.000) |
|  | (0.050) | (0.034) | (0.025) | (0.028) |
| P_C_total_ | 0.021^**^ | 0.000 | 0.008^**^ | 0.019^***^ |
|  | [0.004,0.038] | [-0.008,0.009] | [0.001,0.014] | [0.012,0.027] |
|  | (0.014) | (0.951) | (0.020) | (0.000) |
|  | (0.009) | (0.004) | (0.003) | (0.004) |
| P_C_ward_ | -0.045^***^ | 0.009 | -0.006 | -0.028^***^ |
|  | [-0.074,-0.016] | [-0.006,0.025] | [-0.018,0.006] | [-0.042,-0.014] |
|  | (0.003) | (0.242) | (0.364) | (0.000) |
|  | (0.015) | (0.008) | (0.006) | (0.007) |
| C_total_ | 0.001^***^ | -0.002^***^ | -0.001^***^ | -0.001^***^ |
|  | [0.000,0.002] | [-0.003,-0.001] | [-0.001,-0.000] | [-0.001,-0.000] |
|  | (0.004) | (0.000) | (0.001) | (0.003) |
|  | (0.001) | (0.000) | (0.000) | (0.000) |
| C_PatNum_ | -0.017 | 0.043 | 0.065^***^ | 0.071^***^ |
|  | [-0.099,0.066] | [-0.012,0.099] | [0.025,0.104] | [0.026,0.116] |
|  | (0.694) | (0.125) | (0.001) | (0.002) |
|  | (0.042) | (0.028) | (0.020) | (0.023) |
| day_lag | -0.006^*^ | -0.004 | -0.006^***^ | -0.008^***^ |
|  | [-0.011,0.000] | [-0.011,0.002] | [-0.009,-0.002] | [-0.011,-0.005] |
|  | (0.060) | (0.168) | (0.001) | (0.000) |
|  | (0.003) | (0.003) | (0.002) | (0.002) |
| age | -0.001 | 0.000 | 0.000 | -0.000 |
|  | [-0.005,0.002] | [-0.002,0.003] | [-0.001,0.002] | [-0.002,0.002] |
|  | (0.568) | (0.776) | (0.637) | (0.742) |
|  | (0.002) | (0.001) | (0.001) | (0.001) |
| gender | -0.072 | 0.037 | -0.009 | 0.011 |
|  | [-0.175,0.031] | [-0.036,0.109] | [-0.061,0.043] | [-0.047,0.070] |
|  | (0.172) | (0.321) | (0.739) | (0.698) |
|  | (0.053) | (0.037) | (0.027) | (0.030) |
| loc | 0.001^*^ | -0.003^***^ | -0.003^***^ | -0.002^***^ |
|  | [-0.000,0.003] | [-0.004,-0.002] | [-0.003,-0.002] | [-0.003,-0.001] |
|  | (0.076) | (0.000) | (0.000) | (0.000) |
|  | (0.001) | (0.001) | (0.000) | (0.000) |
| Diagnosis_count | -0.063 | -0.059 | -0.059 | -0.098 |
|  | [-0.310,0.184] | [-0.236,0.117] | [-0.167,0.049] | [-0.217,0.021] |
|  | (0.619) | (0.511) | (0.285) | (0.106) |
|  | (0.126) | (0.090) | (0.055) | (0.061) |
| dept | Yes | Yes | Yes | Yes |
| spec | Yes | Yes | Yes | Yes |
| race | Yes | Yes | Yes | Yes |
| day | Yes | Yes | Yes | Yes |
| mth | Yes | Yes | Yes | Yes |
| constant | 2.065 | -1.942 | -0.733 | 0.026 |
|  | [-0.948,5.077] | [-4.437,0.552] | [-2.802,1.336] | [-2.071,2.124] |
| *N* | 40450 | 40466 | 40466 | 40466 |
| *AIC* | 15770.440 | 28671.394 | 48945.673 | 41767.652 |
| *BIC* | 16682.869 | 29592.474 | 49866.753 | 42688.731 |
| Log Likelihood | -7779.220 | -14228.697 | -24365.837 | -20776.826 |

**Note:** We use the subsample of physicians with the level of resident for this analysis. Coefficients are exponentiated and represent odds ratios. *H_#_* represents habits strength with # as the corresponding habit learning rate. All multivariate models adjusted for the context of the alert, physician's historical exposure to alerts, physician characteristics, patient characteristics, and timing effects. ^***^ indicates *p* < 0.01, ^**^ indicates *p* < 0.05, ^*^ indicates *p* < 0.10.

**Table S10. Fixed Effects Logistic Regression Models for Fellow Subsample**

| Outcome  Predictor | dismiss | dismiss1 | dismiss2 | dismiss3 |  |
| --- | --- | --- | --- | --- | --- |
|  | β coeff.  [95% C.I.]  (P value)  (std.error) | β coeff.  [95% C.I.]  (P value)  (std.error) | β coeff.  [95% C.I.]  (P value)  (std.error) | β coeff.  [95% C.I.]  (P value)  (std.error) |  |
|  |  |  |  |  |  |
| H_0.05_ | 1.591^**^ | 2.285^***^ | 1.831^***^ | 1.584^***^ |  |
|  | [0.372,2.811] | [1.397,3.173] | [1.333,2.329] | [1.010,2.157] |  |
|  | (0.011) | (0.000) | (0.000) | (0.000) |  |
|  | (0.622) | (0.453) | (0.254) | (0.292) |  |
| ward | 0.042 | -0.248^*^ | -0.117 | -0.284^***^ |  |
|  | [-0.299,0.383] | [-0.543,0.047] | [-0.292,0.058] | [-0.475,-0.092] |  |
|  | (0.810) | (0.100) | (0.190) | (0.004) |  |
|  | (0.174) | (0.151) | (0.089) | (0.098) |  |
| P_C_total_ | 0.074 | -0.070^*^ | 0.037 | 0.036 |  |
|  | [-0.031,0.179] | [-0.141,0.001] | [-0.010,0.084] | [-0.018,0.089] |  |
|  | (0.169) | (0.055) | (0.123) | (0.192) |  |
|  | (0.054) | (0.036) | (0.024) | (0.027) |  |
| P_C_ward_ | -0.169^**^ | 0.151^**^ | -0.022 | -0.002 |  |
|  | [-0.330,-0.009] | [0.031,0.271] | [-0.099,0.055] | [-0.087,0.083] |  |
|  | (0.039) | (0.014) | (0.577) | (0.964) |  |
|  | (0.082) | (0.061) | (0.039) | (0.043) |  |
| C_total_ | -0.008 | -0.007 | -0.003 | 0.002 |  |
|  | [-0.021,0.005] | [-0.017,0.003] | [-0.008,0.003] | [-0.005,0.009] |  |
|  | (0.220) | (0.153) | (0.352) | (0.513) |  |
|  | (0.007) | (0.005) | (0.003) | (0.004) |  |
| C_PatNum_ | 0.007 | -0.037 | 0.047 | -0.056 |  |
|  | [-0.345,0.358] | [-0.387,0.314] | [-0.134,0.228] | [-0.253,0.140] |  |
|  | (0.971) | (0.837) | (0.608) | (0.574) |  |
|  | (0.179) | (0.179) | (0.092) | (0.100) |  |
| day_lag | -0.014 | -0.023 | -0.020^**^ | -0.006 |  |
|  | [-0.048,0.020] | [-0.058,0.012] | [-0.036,-0.004] | [-0.023,0.012] |  |
|  | (0.422) | (0.194) | (0.015) | (0.538) |  |
|  | (0.017) | (0.018) | (0.008) | (0.009) |  |
| age | -0.016^**^ | -0.004 | -0.003 | -0.004 |  |
|  | [-0.029,-0.003] | [-0.015,0.007] | [-0.010,0.003] | [-0.011,0.003] |  |
|  | (0.012) | (0.458) | (0.279) | (0.233) |  |
|  | (0.006) | (0.006) | (0.003) | (0.004) |  |
| gender | -0.358^**^ | 0.028 | 0.002 | -0.034 |  |
|  | [-0.694,-0.022] | [-0.268,0.325] | [-0.172,0.176] | [-0.221,0.153] |  |
|  | (0.037) | (0.851) | (0.981) | (0.720) |  |
|  | (0.172) | (0.151) | (0.089) | (0.095) |  |
| loc | -0.002 | -0.006^**^ | -0.001 | -0.002^*^ |  |
|  | [-0.007,0.002] | [-0.011,-0.001] | [-0.003,0.001] | [-0.004,0.000] |  |
|  | (0.310) | (0.026) | (0.153) | (0.056) |  |
|  | (0.002) | (0.003) | (0.001) | (0.001) |  |
| Diagnosis_count | -0.642 | -0.671 | -0.120 | -0.286 |  |
|  | [-1.665,0.382] | [-1.711,0.369] | [-0.533,0.293] | [-0.740,0.167] |  |
|  | (0.219) | (0.206) | (0.570) | (0.215) |  |
|  | (0.522) | (0.531) | (0.211) | (0.231) |  |
| dept | Yes | Yes | Yes | Yes |  |
| spec | Yes | Yes | Yes | Yes |  |
| race | Yes | Yes | Yes | Yes |  |
| day | Yes | Yes | Yes | Yes |  |
| mth | Yes | Yes | Yes | Yes |  |
| constant | 5.621^***^ | -0.326 | 0.715 | 2.060^**^ |  |
|  | [2.677,8.565] | [-2.844,2.192] | [-0.662,2.092] | [0.459,3.662] |  |
| *N* | 3656 | 3695 | 3751 | 3754 |  |
| *AIC* | 1970.652 | 2103.541 | 4849.000 | 4354.314 |  |
| *BIC* | 2541.431 | 2681.511 | 5453.288 | 4964.910 |  |
| Log Likelihood | -893.326 | -958.770 | -2327.500 | -2079.157 |  |

**Note:** We use the subsample of physicians with the level of fellow for this analysis. Coefficients are exponentiated and represent odds ratios. *H_#_* represents habits strength with # as the corresponding habit learning rate. All multivariate models adjusted for the context of the alert, physician's historical exposure to alerts, physician characteristics, patient characteristics, and timing effects. ^***^ indicates *p* < 0.01, ^**^ indicates *p* < 0.05, ^*^ indicates *p* < 0.10.

**Table S11. Fixed Effects Logistic Regression Models for Attending Physician Subsample**

| Outcome  Predictor | dismiss | dismiss1 | dismiss2 | dismiss3 |
| --- | --- | --- | --- | --- |
|  | β coeff.  [95% C.I.]  (P value)  (std.error) | β coeff.  [95% C.I.]  (P value)  (std.error) | β coeff.  [95% C.I.]  (P value)  (std.error) | β coeff.  [95% C.I.]  (P value)  (std.error) |
| H_0.05_ | 1.830^***^ | 1.934^***^ | 2.077^***^ | 1.652^***^ |
|  | [1.008,2.651] | [1.272,2.596] | [1.641,2.512] | [1.184,2.121] |
|  | (0.000) | (0.000) | (0.000) | (0.000) |
|  | (0.419) | (0.338) | (0.222) | (0.239) |
| ward | 0.146 | -0.106 | 0.022 | 0.096 |
|  | [-0.156,0.448] | [-0.363,0.150] | [-0.141,0.185] | [-0.084,0.276] |
|  | (0.343) | (0.417) | (0.794) | (0.294) |
|  | (0.154) | (0.131) | (0.083) | (0.092) |
| P_C_total_ | 0.030 | 0.038 | 0.027 | 0.077^***^ |
|  | [-0.065,0.124] | [-0.024,0.100] | [-0.017,0.071] | [0.024,0.130] |
|  | (0.539) | (0.234) | (0.223) | (0.004) |
|  | (0.048) | (0.032) | (0.022) | (0.027) |
| P_C_ward_ | -0.201^**^ | 0.009 | 0.009 | -0.083^*^ |
|  | [-0.377,-0.026] | [-0.103,0.121] | [-0.071,0.088] | [-0.178,0.012] |
|  | (0.025) | (0.870) | (0.832) | (0.086) |
|  | (0.090) | (0.057) | (0.041) | (0.049) |
| C_total_ | -0.001 | -0.006^**^ | -0.003^*^ | -0.001 |
|  | [-0.009,0.006] | [-0.011,-0.000] | [-0.007,0.001] | [-0.006,0.003] |
|  | (0.747) | (0.046) | (0.095) | (0.506) |
|  | (0.004) | (0.003) | (0.002) | (0.002) |
| C_PatNum_ | 0.048 | -0.180 | 0.144 | 0.243^**^ |
|  | [-0.269,0.364] | [-0.499,0.139] | [-0.031,0.320] | [0.044,0.442] |
|  | (0.768) | (0.268) | (0.106) | (0.017) |
|  | (0.162) | (0.163) | (0.089) | (0.102) |
| day_lag | -0.002 | 0.006 | 0.001 | -0.003 |
|  | [-0.022,0.018] | [-0.011,0.022] | [-0.008,0.011] | [-0.012,0.007] |
|  | (0.862) | (0.498) | (0.779) | (0.597) |
|  | (0.010) | (0.008) | (0.005) | (0.005) |
| age | -0.000 | 0.005 | -0.004 | 0.001 |
|  | [-0.009,0.009] | [-0.004,0.014] | [-0.009,0.002] | [-0.005,0.006] |
|  | (0.976) | (0.298) | (0.164) | (0.802) |
|  | (0.005) | (0.005) | (0.003) | (0.003) |
| gender | -0.113 | -0.172 | -0.116 | -0.155^*^ |
|  | [-0.374,0.148] | [-0.425,0.081] | [-0.267,0.035] | [-0.315,0.004] |
|  | (0.395) | (0.183) | (0.133) | (0.056) |
|  | (0.133) | (0.129) | (0.077) | (0.081) |
| loc | 0.002 | -0.002 | -0.002^***^ | -0.001^*^ |
|  | [-0.001,0.005] | [-0.006,0.001] | [-0.004,-0.001] | [-0.003,0.000] |
|  | (0.156) | (0.224) | (0.005) | (0.097) |
|  | (0.002) | (0.002) | (0.001) | (0.001) |
| Diagnosis_count | 0.797^**^ | -0.261 | 0.232 | 0.300^*^ |
|  | [0.070,1.524] | [-0.869,0.348] | [-0.079,0.543] | [-0.048,0.648] |
|  | (0.032) | (0.401) | (0.143) | (0.091) |
|  | (0.371) | (0.310) | (0.159) | (0.177) |
| dept | Yes | Yes | Yes | Yes |
| spec | Yes | Yes | Yes | Yes |
| race | Yes | Yes | Yes | Yes |
| day | Yes | Yes | Yes | Yes |
| mth | Yes | Yes | Yes | Yes |
| constant | 0.126 | -3.123^***^ | -1.997 | -1.411 |
|  | [-3.850,4.103] | [-4.673,-1.573] | [-4.769,0.775] | [-4.249,1.427] |
| *N* | 5313 | 5255 | 5430 | 5448 |
| *AIC* | 2957.874 | 2987.051 | 6560.140 | 6045.617 |
| *BIC* | 3589.353 | 3564.941 | 7213.510 | 6712.520 |
| Log Likelihood | -1382.937 | -1405.525 | -3181.070 | -2921.808 |

**Note:** We use the subsample of physicians with the level of attending physician for this analysis. Coefficients are exponentiated and represent odds ratios. *H_#_* represents habits strength with # as the corresponding habit learning rate. All multivariate models adjusted for the context of the alert, physician's historical exposure to alerts, physician characteristics, patient characteristics, and timing effects. ^***^ indicates *p* < 0.01, ^**^ indicates *p* < 0.05, ^*^ indicates *p* < 0.10.
