# Supplementary material for: Habit and Automaticity in Medical Alert Override: Cohort Study
Source: J Med Internet Res. 2022 Feb 16;24(2):e23355. doi: 10.2196/23355 (PMC8892274; doi:10.2196/23355)
Supplement: Multimedia Appendix 2 [file jmir_v24i2e23355_app2.docx]

Multimedia Appendix 2. Fixed effects logistics regression results for dismissal.

| **Outcome**  **Predictor** | ***dismiss*** | ***dismiss*** | ***dismiss*** | ***dismiss1*** | ***dismiss1*** | ***dismiss1*** |
| --- | --- | --- | --- | --- | --- | --- |
|  | *β* coeff.  [95% C.I.]  (*P* value)  (std.error) | *β* coeff.  [95% C.I.]  (*P* value)  (std.error) | *β* coeff.  [95% C.I.]  (*P* value)  (std.error) | *β* coeff.  [95% C.I.]  (*P* value)  (std.error) | *β* coeff.  [95% C.I.]  (*P* value)  (std.error) | *β* coeff.  [95% C.I.]  (*P* value)  (std.error) |
| *Habit Strength*  *H_0.01_* | 2.195***  [1.767,2.623]  (0.000)  (0.220) |  |  | 1.663***  [1.376,1.949]  (0.000)  (0.147) |  |  |
| *Habit Strength*  *H_0.05_* |  | 2.506***  [2.312,2.700]  (0.000)  (0.100) |  |  | 1.391***  [1.231,1.551]  (0.000)  (0.082) |  |
| *Habit Strength*  *H_0.1_* |  |  | 2.784***  [2.620,2.948]  (0.000)  (0.085) |  |  | 1.540***  [1.379,1.701]  (0.000)  (0.083) |
| **Outcome**  **Predictor** | ***dismiss2*** | ***dismiss2*** | ***dismiss2*** | ***dismiss3*** | ***dismiss3*** | ***dismiss3*** |
|  | *β* coeff.  [95% C.I.]  (*P* value)  (std.error) | *β* coeff.  [95% C.I.]  (*P* value)  (std.error) | *β* coeff.  [95% C.I.]  (*P* value)  (std.error) | *β* coeff.  [95% C.I.]  (*P* value)  (std.error) | *β* coeff.  [95% C.I.]  (*P* value)  (std.error) | *β* coeff.  [95% C.I.]  (*P* value)  (std.error) |
| *Habit Strength*  *H_0.01_* | 1.236***  [1.054,1.419]  (0.000)  (0.093) |  |  | 1.197***  [0.994,1.400]  (0.000)  (0.104) |  |  |
| *Habit Strength*  *H_0.05_* |  | 1.467***  [1.364,1.569]  (0.000)  (0.052) |  |  | 1.439***  [1.331,1.547]  (0.000)  (0.055) |  |
| *Habit Strength*  *H_0.1_* |  |  | 1.753***  [1.653,1.852]  (0.000)  (0.051) |  |  | 1.728***  [1.627,1.829]  (0.000)  (0.052) |

**Note:** Coefficients are exponentiated and represent odds ratios*.* *H_#_* represents habit strength with # as the corresponding habit learning rate. All multivariate models were adjusted for the context of the alert, physician’s historical exposure to alerts, physician characteristics, patient characteristics, and timing effects. Full regression results are available in supplementary materials. ^***^ indicates *p* < 0.01.
